# Super‐Resolution Axial Imaging for Quantifying Piconewton Traction Forces in Live Cells

**DOI:** 10.1002/anie.202506864

**Published:** 2025-08-18

**Authors:** Dong‐Xia Wang, José Ignacio Gallea, De‐Ming Kong, Jörg Enderlein, Tao Chen

**Affiliations:** ^1^ Third Institute of Physics – Biophysics Georg August University Friedrich‐Hund‐Platz 1 37077 Göttingen Germany; ^2^ State Key Laboratory of Medicinal Chemical Biology Tianjin Key Laboratory of Biosensing and Molecular Recognition Research Centre for Analytical Sciences College of Chemistry Nankai University Tianjin 300071 P.R. China; ^3^ Department of Molecular Biology Princeton University Princeton NJ 08540 USA; ^4^ Cluster of Excellence “Multiscale Bioimaging: from Molecular Machines to Networks of Excitable Cells” (MBExC) Universitätsmedizin Göttingen Robert‐Koch‐Str. 40 37075 Göttingen Germany

**Keywords:** Fluorescence lifetime imaging microscopy, Mechanochemical biology, Metal‐induced energy transfer imaging, Molecular tension probe, Super‐resolution imaging

## Abstract

Cell mechanics play a pivotal role in regulating numerous biological processes. Although super‐resolution microscopy enables the imaging of cellular forces in the lateral dimension with sub‐10‐nm resolution, achieving comparable resolution along the axial dimension remains a significant challenge. Here, we introduce metal‐induced energy transfer (MIET)‐based tension probe microscopy (MIET‐TPM), a technique for mapping cellular mechanical forces with nanometer precision in the axial direction. This approach combines the nanometer spatial resolution of MIET imaging with the piconewton sensitivity of DNA‐hairpin‐based molecular tension probes (MTPs), enabling the simultaneous observation of both the plasma membrane and force‐exerting molecules in the axial dimension. Using MIET‐TPM, we mapped axial integrin tension within focal adhesions and podosomes, alongside their corresponding plasma membrane height profiles, offering detailed insights into the nanoscale structures and mechanisms involved in force transmission. Notably, MIET‐TPM can be implemented on any fluorescence microscopy setup without hardware modifications, making it a versatile and accessible tool that promises to become an integral part of future cellular mechanobiology analysis.

## Graphical abstract

We introduce metal‐induced energy transfer‐based tension probe microscopy (MIET‐TPM), a hybrid imaging approach that combines MIET with DNA‐based molecular tension probes to map piconewton cellular traction forces with nanometer axial resolution. This method enables simultaneous visualization of integrin forces and membrane topography in live cells.

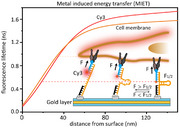

## Introduction

Cell mechanics play a crucial role in regulating various biological processes, such as immune recognition, blood coagulation, cell migration, and differentiation.^[^ ^1^, ^2^, ^3^ ^]^ Structures responsible for force transmission, including filopodia, focal adhesions (FAs), podosomes, and the cytoskeleton, are dynamically organized at the nanoscale in all dimensions.^[^ ^4^, ^5^ ^]^ To gain a deeper understanding of how mechanical forces interact with biochemical signaling pathways, it is essential to develop advanced methods capable of mapping the distribution of forces within living cells. Molecular tension probes (MTPs) have been developed to measure receptor forces with piconewton (pN) sensitivity.^[^ ^6^, ^7^ ^]^ These probes consist of an extendable “spring‐like” element, such as polyethylene glycol or DNA hairpins, flanked by a fluorophore and a quencher, and with one end immobilized on a surface. When receptor forces are applied to the other end of the probe, the “spring” extends, causing the fluorophore and quencher to separate, which in turn increases fluorescence. This change in fluorescence can be detected using various microscopy techniques, enabling the mapping of receptor forces in cellular adhesion structures such as podosomes,^[^ ^8^, ^9^ ^]^ FAs,^[^ ^10^, ^11^ ^]^ or E‐cadherin complexes.^[^ ^12^ ^]^

Current fluorescence techniques, such as total internal reflection fluorescence microscopy, confocal microscopy, and structured illumination microscopy, are limited to mapping forces in the lateral dimension.^[^ ^13^, ^14^ ^]^ Even with super‐resolution techniques like localized super‐resolution microscopy like DNA points accumulation for imaging in nanoscale topography (DNA‐PAINT), which can achieve resolutions of a few nanometers in the lateral direction,^[^ ^15^, ^16^ ^]^ the resolution in the axial dimension is limited to tens of nanometers.^[^ ^15^ ^]^ However, because cells apply axial forces to the MTPs to separate the fluorophore–quencher pair, accurately characterizing tension forces along the axial direction is more critical than in the lateral plane. Methods such as astigmatic traction force microscopy (aTFM) enable 3D super resolution force mapping and have improved axial resolution to 22 nm.^[^ ^17^ ^]^ Importantly, achieving sub‐10‐nm axial resolution will allow for the direct observation of vertical force transmission through discrete molecular linkages–such as the integrin–talin–actin axis and nanoscale membrane deformations that occur during mechanotransduction.^[^ ^18^, ^19^ ^]^ This level of precision is crucial for elucidating how mechanical signals are spatially organized and processed at the molecular scale.^[^ ^20^ ^]^ Despite this need, no current technique permits mapping of cellular forces with sub‐10‐nm resolution in the axial direction.

Metal‐induced energy transfer (MIET) imaging/spectroscopy is a recently developed technique that enables precise determination of the axial position of a fluorescent single molecule above a metal film with a precision of approximately 3 nm.^[^ ^21^, ^22^, ^23^, ^24^ ^]^ This method has been applied to a wide range of systems, from whole cells to organelles.^[^ ^21^, ^22^, ^23^, ^24^, ^25^, ^26^, ^27^, ^28^, ^29^ ^]^ Furthermore, MIET exhibits significant versatility and has been integrated with other techniques to expand its applications. For instance, its combination with fluorescence correlation spectroscopy has enabled the study of membrane dynamics,^[^ ^30^, ^31^ ^]^ while its integration with single‐molecule localization microscopy has facilitated three‐dimensional isotropic resolution imaging of microtubules and clathrin.^[^ ^32^ ^]^

In this work, we introduce metal‐induced energy transfer‐based tension probe microscopy (MIET‐TPM), a novel methodology that overcomes current limitations by enabling the measurement of axial forces with nanometer precision. MIET‐TPM integrates the nanometer‐scale spatial resolution of MIET with the piconewton (pN) force sensitivity of MTPs, allowing for precise detection of pN‐level axial forces generated by living cells while maintaining high axial resolution.

## Results and Discussion

**Figure 1:**
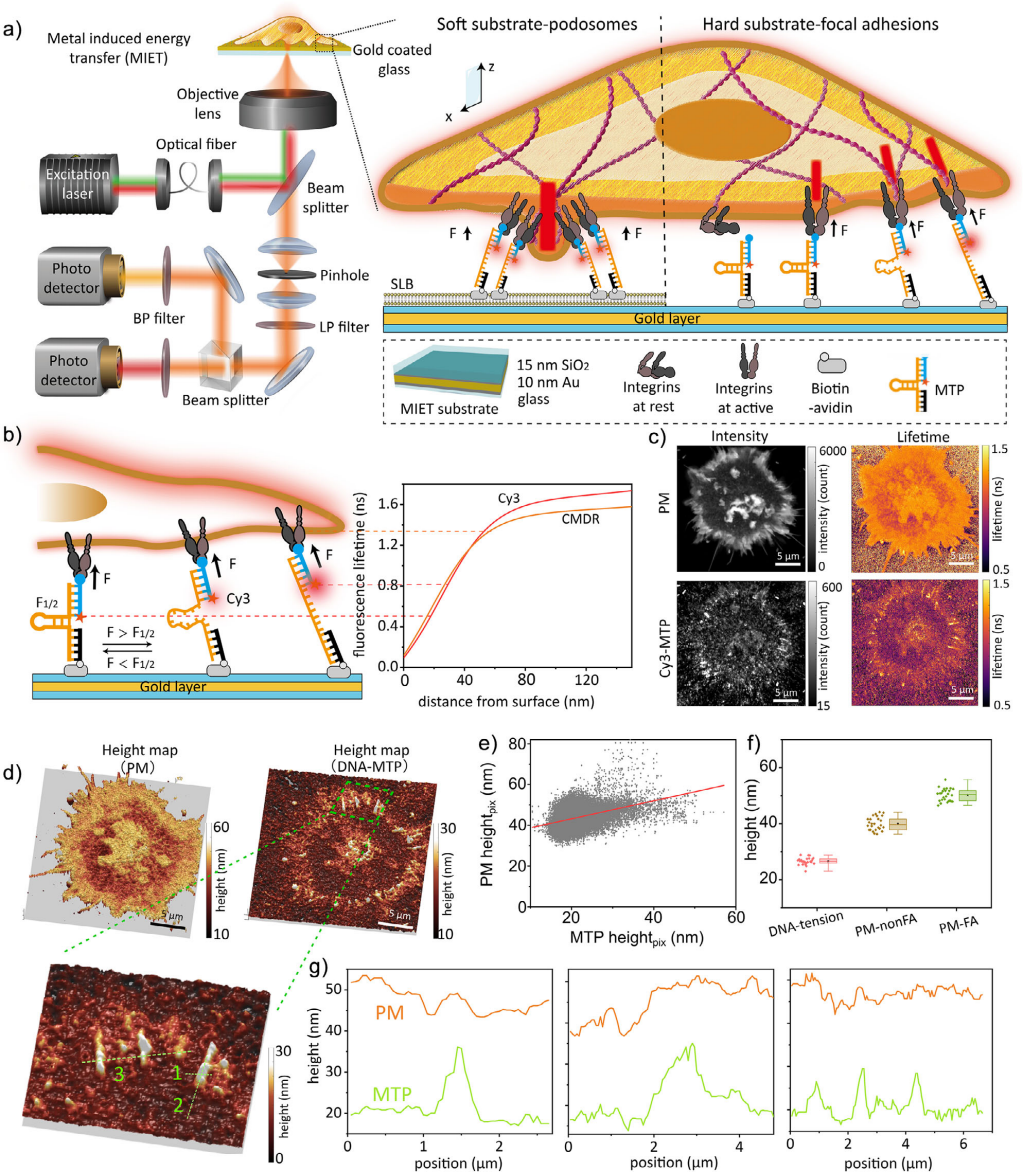
MIET‐TPM measurement on FA. a) Left: diagram showing the MIET‐TPM setup for two‐color measurement. Cos7 cells are seeded on the MTP‐modified MIET substrate, which consists of a 10‐nm gold film sandwiched between a 15‐nm silica layer and a glass coverslip; Right: schematic illustrating integrin‐involved adhesion structures: podosome and FA. b) Working principle of MIET‐TPM, which includes an anchor strand immobilized on the MIET substrate, a hairpin strand that unfolds under sufficient tension and a ligand stand presenting an adhesive peptide. The right panel shows the calculated fluorescence lifetime‐versus‐distance curves (MIET curves) for fluorophores Cy3 and CMDR fluorophores, respectively. The calculation details provided in Table S2. c) The fluorescence intensity images and corresponding lifetime images used to determine the heights of Cy3 and CMDR, respectively. d) Reconstructed height maps for MTP and PM. The bottom panel shows the enlarged area. e) Pixel‐wise scatter plot of PM height versus MTP height across the entire cell. Each point represents an individual pixel. The red line represents the linear regression fit, illustrating the overall trend between PM and MTP heights. f) Box plot of the height values for MTP, PM without FA (PM‐nonFA), and PM with FA (PM‐FA). Box plots show the 25th–75th quantiles (box), median (solid line), mean (black dot), and whiskers (minima to maxima). *n* = 26 independent cells. g) Linescan analysis for the height profiles of PM and MTP.

MIET imaging/spectroscopy uses the phenomenon that a fluorescence emitter, when brought close to a metal surface, transfers its excited‐state energy to surface plasmons (collective oscillations of free electrons) of the metal film.^[^ ^21^, ^30^, ^33^ ^]^ This energy transfer is distance‐dependent, resulting in the modulation of both fluorescence lifetime and intensity based on the proximity between the emitter and the metal surface. This mechanism is analogous to Förster resonance energy transfer (FRET),^[^ ^34^ ^]^ which involves energy transfer from a donor to an acceptor fluorophore. The measured fluorescence lifetime can then be converted into a precise distance between the emitter and the metal surface using semiclassical quantum‐electrodynamic theory.^[^ ^35^ ^]^ Additionally, due to the broad absorption spectra of metal, the energy transfer from a fluorescent molecule to the metal film occurs with high efficiency across the visible spectrum, allowing any dye within this spectral range to exhibit the effect. This feature facilitates the co‐localization of multiple fluorophores through MIET (Figure 1a).

Matrix‐activated integrins can form different adhesion structures of fibroblasts depending on the substrate:^[^ ^8^, ^36^ ^]^ FAs when spread on rigid surfaces and podosome‐like adhesions on Arg‐Gly‐Asp peptide (RGD)‐modified fluid lipid surfaces. We employed MIET‐TPM to study both types of adhesion structures. To map forces along the axial direction, we utilized MIET in conjunction with a widely recognized DNA‐based hairpin MTP. One end of the MTP was modified with an RGD molecule, designed to activate and bind integrin on the cell surface. The other end was modified with a biotin molecule to anchor the MTP to an avidin‐modified surface or an avidin‐modified supported lipid bilayer (SLB) on a MIET substrate. The MIET substrate comprises a 10‐nm gold film sandwiched between a 15‐nm silica layer and a glass coverslip (Figure 1a). Since the gold film acts as a quencher in MTPs, no additional quencher molecule is required.

When integrin‐mediated forces exceed the $F_{1/2}$, the equilibrium force at which 50% of hairpins unfold, the dye lifts from the surface, leading to an increase in fluorescence lifetime and intensity (Figure 1b). In contrast, hairpins experiencing forces lower than $F_{1/2}$ remain folded and positioned closer to the surface, exhibiting a shorter fluorescence lifetime and intensity. We followed previous reports using DNA‐MTPs with $F_{1/2}$ values of 4.7 or 19 pN^[^ ^11^ ^]^ (Table S1), both of which are below the reported force threshold for initial integrin adhesion,^[^ ^37^ ^]^ estimated to be ∼ 40 pN. Additionally, by labeling the plasma membrane (PM) with a separate fluorophore, we reconstructed the height profile of the basal PM decorated with MTPs, providing insights into how adhesion structures affect the basal membrane.

Initially, we tested our ability to measure the traction forces generated by FAs in Cos7 cells, as mechanical forces regulate their activation and clotting.^[^ ^5^ ^]^ Cos7 cells were seeded on Cy3‐MTPs‐modified MIET substrate, which produced a robust signal corresponding to integrins applying forces greater than 4.7 pN ($F_{1/2}$). The PM was labeled with a commercial dye, CellMask Deep Red (CMDR), to report its height. Control experiments confirmed that no FRET occurs between Cy3‐MTPs and CMDR due to the significant distance between the Cy3 position and the PM (Figure S5, Note S2). This setup enabled us to simultaneously measure Cy3‐MTPs and PM height using two detectors.

We imaged the sample using a laser‐scanning confocal microscope (CLSM) equipped with time‐correlated single‐photon counting (TCSPC) to perform fluorescence lifetime measurements. To accurately determine the lifetime values for both the fluorophores in the MTPs and the PM, we scanned individual cells to accumulate sufficient signals for reliable fluorescence decay fitting. TCSPC curves were generated for each pixel and fitted with a multiexponential decay model (Note S3), yielding a mean fluorescence lifetime for each pixel (50 nm × 50 nm). These lifetime images were subsequently converted to height maps above the silica surface using fluorophore‐specific MIET calibration curves.

Figure 1c shows the measured fluorescence intensity and lifetime images for MTPs and PM in a single Cos7 cell after 20 min of incubation. The cell edges exhibit higher intensity (approximately 10 times stronger than the background) and longer lifetime for both MTPs and PM, indicating a higher density of hairpin unfolding at these regions. This observation is consistent with previous reports using MTPs based on fluorophore–quencher pairs.^[^ ^11^, ^38^ ^]^ We further constructed 3D height maps for MTPs and PM (Figure 1d) from the lifetime images. FA areas are clearly identifiable by their elevated MTP height. We calculated the average height for each FA region, focusing on areas with a signal‐to‐noise ratio (SNR) greater than 10 (Figure S6). The mean height of the FAs is approximately 26 nm, which is ∼ 9 nm higher than the height of closed MTPs (17.6 nm, Figure S7, Note S4).

In MIET measurements, the axial localization precision is determined by the number of photons used in the fluorescence lifetime fitting. For DNA‐MTPs, the minimum photon count per pixel is approximately 400 (Figures S8 and S9). Fitting with 400 photons results in a height uncertainty of around 2 nm (Note S3). For the PM height, the minimum photon count per pixel is approximately 1500, which produces a height uncertainty of less than 1 nm. It is important to note that the height values obtained for each pixel is a spatial average over the excitation focus area, encompassing both folded and unfolded MTPs. However, in regions with high fluorescence intensity (FAs with SNR > 10), the signal is dominated by unfolded MTPs, and thus the measured fluorescence lifetime primarily reflects the height of these unfolded MTPs (Figure S10).

We further performed a linescan analysis on both the FA and PM within the same area (Figure 1d,g). Interestingly, a positive local trend was observed: regions with higher FAs tend to correspond to higher PM heights. This same trend was also seen in the 19 pN MTPs (Figure S11). To better quantify the relationship between integrin tension and PM topography, we performed a pixel‐wise correlation analysis between MTP height and PM height across the entire cell. As shown in Figure 1e, a moderate positive correlation was observed (Pearson's *r* = 0.36), indicating a local tendency for regions with higher integrin‐mediated tension to coincide with upward displacement of the basal PM. It is important to note that the relatively low correlation may be attributed to the structural heterogeneity of FAs and the inherent complexity of PM deformation dynamics. Figure 1f presents the statistical data from 26 cells using 4.7 pN MTPs. Our results demonstrated distinct structures for the FA and PM: the unfolded MTPs have a mean height of 26.6 ± 1.3 nm (mean ± SD, *N* = 26), while the FA regions of the PM had a mean height of 50.2 ± 2.3 nm, and the non‐FA regions of the PM showed a mean height of 40.0 ± 2.4 nm.

From the MTP height images, we observed that the heights of the unfolded 4.7 pN MTPs varied wildly, ranging from 20 to 40 nm (Figure S11g), indicating heterogeneity in the tension forces. Theoretically, the maximum height for fully stretched 4.7 pN MTPs should be approximately 35 nm if the DNA chain is fully extended and remains upright on the surface. However, about 5% of the FA regions displayed heights greater than 35 nm, which could be attributed to DNA overstretching under constant tension.^[^ ^39^ ^]^ It has been reported that overstretching can induce dehybridization of short DNA sequences.^[^ ^40^, ^41^, ^42^ ^]^ For example, overstretching‐induced dehybridization has been observed at forces as low as 16 pN for an 18‐bp probe with 40% GC content at 37 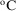.^[^ ^43^ ^]^ Based on comparable parameters, the estimated dehybridization force for our DNA hairpin‐based MTP lies in the range of 30–45 pN, which partially overlaps with the range of forces exerted by single integrins (2–40 pN).^[^ ^44^, ^45^, ^46^ ^]^ Therefore, it is reasonable to hypothesize that a small fraction of the MTPs ( 5%, showing elevated heights) may experience forces sufficient to induce overstretching and partial dehybridization.

**Figure 2:**
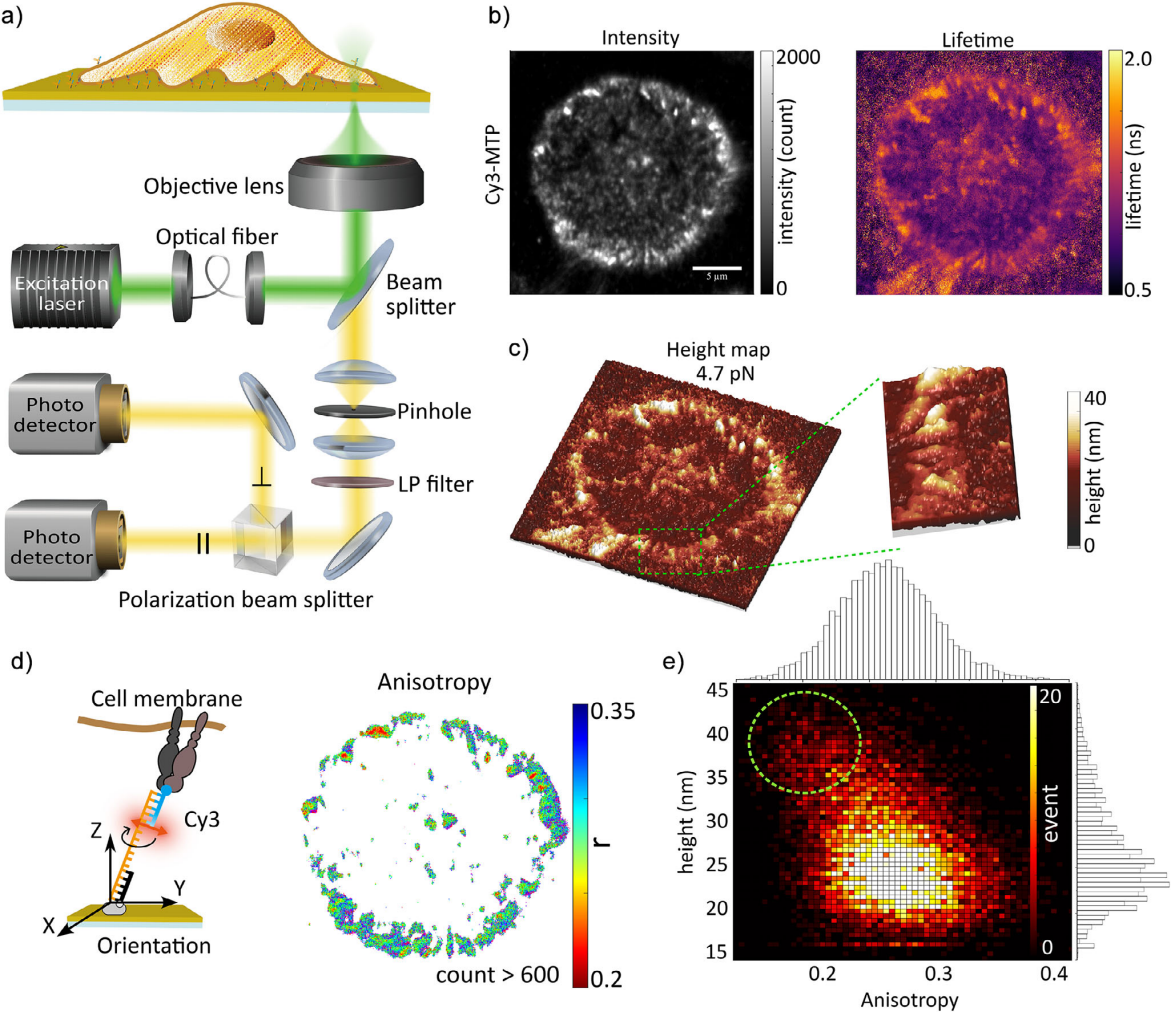
MIET‐anisotropy measurement on FA. a) Diagram illustrating the MIET‐anisotropy measurement. The Cy3 or FAM on the MTP is excited by polarized light and the emission light is separated based on its polarization using a polarization beam splitter into two detectors. b) Fluorescence intensity image and lifetime image of a cell on 4.7 pN Cy3‐MTP‐modifed MIET substrate. c) The corresponding height map. d) Left: Illustration showing the Cy3 orientation perpendicular to the long axis of the tension probe. Right: Calculated anisotropy image of Cy3 on MTP. e) 2D histogram depicting the relationship between the height and anisotropy for Cy3‐modified MTP.

To further investigate this hypothesis, we performed anisotropy measurements on Cy3‐MTPs on MIET substrates, which report the rotational motion of the dye molecule during its excited‐state lifetime.^[^ ^47^ ^]^ In anisotropy measurements, polarized light is used to excite the fluorophore, and the emitted light is separated by polarization using a polarization beam splitter (Figure 2a). The polarized emission is then directed onto two detectors, allowing for the calculation of fluorescence anisotropy, r, from the intensities detected by these two detectors (Figure S12). Cy3 dye can stack against the nucleobases of the DNA duplex, perpendicular to its long axis, which is essential for maintaining a fixed dye orientation relative to the biomolecule, enabling accurate determination of its orientation.^[^ ^48^, ^49^ ^]^

Figure 2 shows the anisotropy measurements for a single cell on MTP‐MIET substrate. The formation of FAs is evident from the intensity, lifetime, and height images (Figure 2b). As expected, we observed high anisotropy (mean r∼ 0.27 for the FAs) in the anisotropy image (Figure 2c), indicating that the rotation of Cy3 is restricted due to stacking on the DNA duplex. Interestingly, a bias toward lower anisotropy values was found at greater heights in the 2D height‐anisotropy histogram (Figure 2d,e). The lower anisotropy suggests increased flexibility of the fluorophore, indicating a reduction in the stacking of Cy3 on the DNA duplex at higher elevations. This increased flexibility is likely caused by DNA overwinding or overstretching‐induced DNA dehybridization when stretched.^[^ ^43^ ^]^ To demonstrate that the observed anisotropy arises from Cy3 stacking on DNA, we labeled the 4.7 pN MTP with another fluorophore, FAM, and measured its anisotropy (Figure S13). FAM is known not to stack on DNA and exhibits rapid rotational motion.^[^ ^50^ ^]^ As expected, the anisotropy of the FAM‐labeled MTP was lower than that of the Cy3‐labeled MTP, and no bias effect was observed in the 2D height‐anisotropy histogram (Figure S13b). Additionally, no bias effect was observed for probes using 19 pN Cy3‐MTPs (Figure S14), as the 19 pN MTP has a longer DNA stretch length in the unfolded state (∼ 45 nm). These MIET‐anisotropy measurements provide further insight into the force structures of FAs using MTPs, suggesting that caution is necessary when using DNA for orientation measurements, particularly under varying force conditions.

To further validate that MTP unfolding and anisotropy signals are induced by cellular forces, we treated cells with latrunculin A to disrupt actin polymerization and reduce integrin‐mediated traction. Following treatment, the FA area showed a noticeable decrease (Figure S15), supporting the conclusion that these readouts are mechanically regulated.

**Figure 3:**
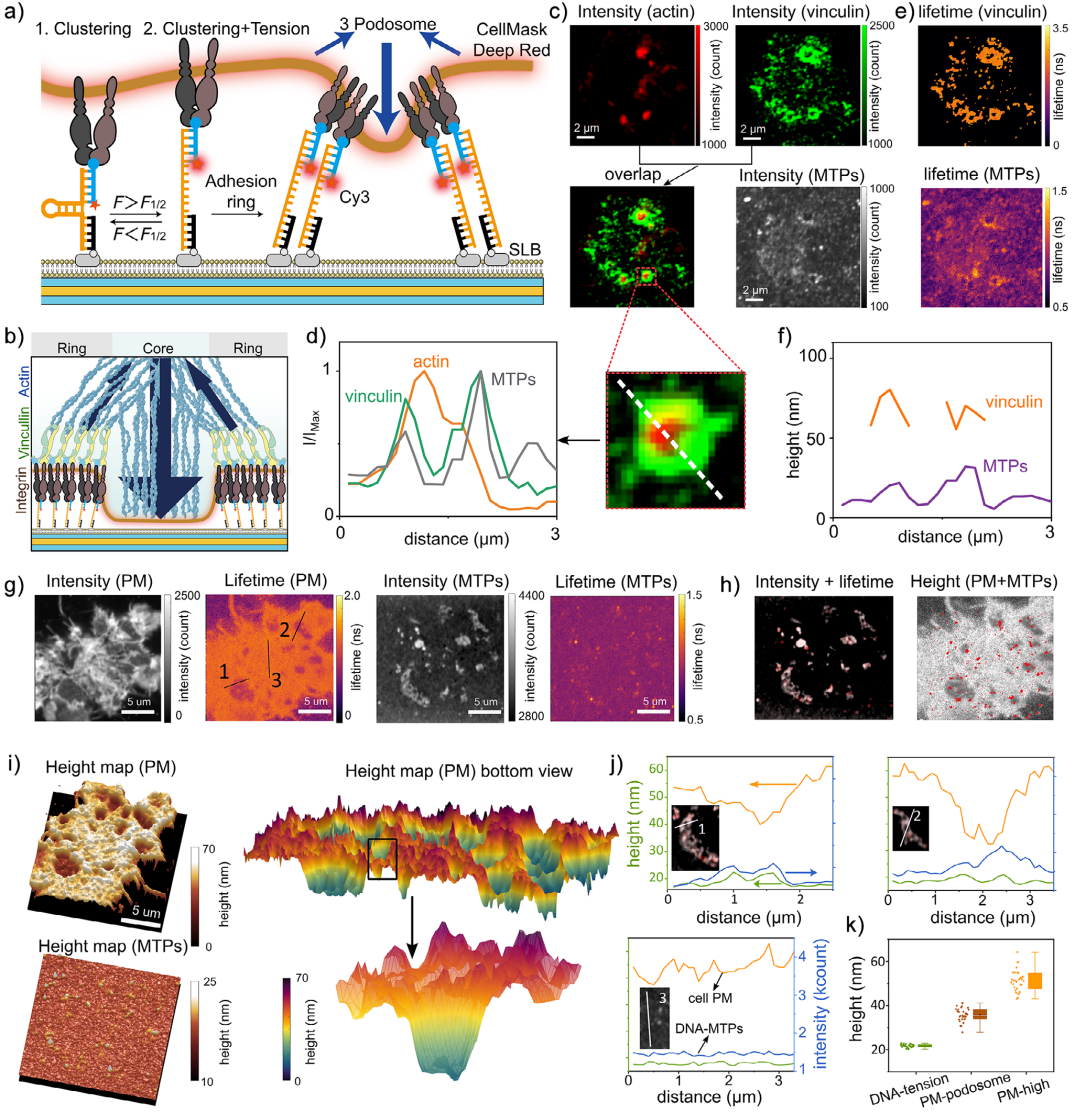
MIET‐TPM measurement on podosomes. a) Diagram illustrating the formation of podosomes in NIH 3T3 fibroblasts. The cells are seeded on the MTP‐modified SLB, which is supported on a MIET substrate. b) Schematic of a single podosome at the cell–SLB interface. c) Fluorescence intensity images for the Phalloidin‐Alexa Fluor 647, Vinculin Antibody‐Alexa Fluor 488, and MTP‐Cy3. Bottom shows the overlap of F‐action image with vinculin image. d) Normalized linescan analysis for the fluorescence intensity of the podosome in (c). Green, orange, and grey lines represent vinculin, actin, and MTPs, respectively. e) The corresponding calculated fluorescence lifetime images for vinculin and Cy3, respectively. f) Corresponding linescan analysis for the height images of the podosome in (e). Purple and orange lines represent MTPs and vinculin, respectively. g–j) MIET‐TPM measurement of colocalization of PM and MTPs on podosomes. g) Fluorescence intensity images and corresponding lifetime images for MTP‐Cy3 and CMDR, respectively. h) Left: Overlay of the MTP intensity image with MTP lifetime image; right: Overlay of the MTP height image with PM height image. i) The reconstructed height maps for MTP and PM. The PM heihgt maps are shown in top view (left) and bottom view (right). j) Linescan analysis for the lines marked in (c). k) Box plot of the height values for MTP, PM‐podosome, and PM without podosome (PM‐high). Box plots show the 25th–75th quantiles (box), median (solid line), mean (black dot), and whiskers (minima to maxima). *n* = 30 independent cells.

Next, we applied MIET‐TPM to map podosome‐like adhesions formed by fibroblasts (Figure 3a). It has been reported that fibroblasts form podosomes when cultured on RGD‐modified membranes,^[^ ^36^ ^]^ and that integrins in fibroblasts can exert ring‐like tensile forces in podosomes on RGD‐modified SLBs (Figure 3b).^[^ ^8^ ^]^ To investigate this with MIET‐TPM, we tethered RGD peptides to the SLB via DNA oligonucleotides and cultured NIH 3T3 fibroblasts on these substrates. Consistent with previous findings,^[^ ^8^, ^36^, ^51^ ^]^ we observed the formation of ring‐like adhesions after 1 h of incubation (Figure 3c). These adhesions were rich in actin, excluded the RGD‐MTP ligand, and were surrounded by vinculin (Figure 3c,d)—a characteristic signature of podosomes. Therefore, we refer to these structures as podosomes throughout the manuscript. In addition to fluorescence intensity imaging, we also obtained lifetime images for both vinculin and MTPs (Figure 3e). Since the fluorescence of both fluorophores is quenched by the MIET effect, we could calculate their axial positions. As shown in Figure 3f, vinculin localized at a significantly higher axial position than MTPs, consistent with its expected intracellular positioning above the plasma membrane. However, since vinculin resides outside the optimal axial working range of MIET (approximately 5–70 nm for most fluorophores), the accuracy of its height determination is lower than that of MTPs (Figure S16, Note S5).

Further, we applied MIET‐TPM to co‐map the MTPs and the PM in living cells (Figure 3g–j). Again, we observed ring‐like structures in the MTPs fluorescence intensity images, and the corresponding podosomes exhibited lower heights in the PM height maps. We found that regions with longer lifetimes in the MTP lifetime image correlated with higher intensities in the MTP intensity image. However, the ring‐like structures were difficult to discern in the MTP lifetime or height images (Figure 3g,h), likely due to the low SNR. In the podosome measurements, the SNR was approximately 2, which led to an underestimation of lifetime values (Note S6, Figure S10). The low SNR may result from factors such as fluorophore diffusion, minimal height increases, and a low percentage of open MTPs.

To confirm that the observed increase in lifetime is due to MTPs unfolding, we conducted a control experiment in which cells were seeded on the SLBs that co‐presented Cy3‐modified MTPs lacking RGD and RGD‐modified MTPs lacking Cy3 (Figure S17). Interestingly, even without the RGD in the Cy3‐modified MTPs, increased intensity was observed in the MTPs intensity image, but no corresponding increase in lifetime was detected, indicating that the Cy3‐MTPs remained folded. Nevertheless, podosome formation was confirmed through PM measurements. The elevated MTP signal intensity observed near podosomes likely result from local confinement between the plasma membrane and the SLB, which restricts lateral diffusion of the MTPs (Figure S18) and leads to local accumulation. Additional contributions may arise from non‐specific interactions between the DNA backbone and membrane‐associated proteins or lipids.

Importantly, the podosome structures were clearly visible in the PM height profiles (Figure 3i). A negative correlation between the unfolding MTPs and PM was observed in the podosome measurements, as shown by the linescan analysis (Figure 3j). Podosomes exhibited lower PM height, which consistently corresponded to higher DNA probe heights and greater brightness. Statistical analysis of 30 cells revealed that the unfolding MTPs had a mean height of 21.7 ± 0.7 nm, the podosome‐associated PM (podosome‐PM) had a mean height of 35.8 ± 3.1 nm, and the non‐podosome PM areas had a mean height of 51.3 ± 4.9 nm (Figure 3k). Control experiments showed that the folded MTPs on SLBs had a mean height of 17.1 ± 1 nm from the surface (Figure S7), indicating only a 4.6 nm increase in height after MTP unfolding.

It is important to note that, due to the low SNR, the actual height of MTPs is likely higher than the measured values. The negative correlation observed is explained by the fact that the core of the podosome structure, where the podosome‐PM height is measured, lacks integrins;^[^ ^8^, ^52^ ^]^ instead, the forces are generated by integrins distributed around the ring of the podosome. To determine the PM height at the podosome rim, we calculated the PM height for areas displaying only longer lifetimes in the MTP lifetime image. The mean height for this area (ring‐podosome PM) is 44.0 ± 7.2 nm, higher than the core‐podosome PM, although still lower than the non‐podosome PM. Measurements using 19 pN MTPs also showed the same negative correlation between the unfolded MTPs and podosome‐PM, suggesting that forces generated within podosomes exceed 19 pN (Figure S19).

## Conclusion

Current techniques for mapping cellular forces are predominantly focused on the lateral plane, while previous efforts to resolve forces along the axial direction have been constrained by limited resolution, typically on the order of tens of nanometers.^[^ ^15^ ^]^ MIET‐TPM offers a complementary approach, achieving nanometer‐scale precision (∼ 2 nm) in resolving cellular forces along the axial direction. Demonstrating its capabilities, MIET‐TPM was used to map integrin tension in FAs and podosomes, revealing distinct structural differences between these adhesion complexes. Despite involving the same force‐bearing molecules, FAs exhibited a positive height correlation between the MTP and the PM, while podosomes showed a negative correlation, reflecting their unique structural and functional characteristics. Furthermore, anisotropy measurements combined with MIET‐TPM showed that partial DNA within the MTP molecule becomes overstretched under integrin tension, offering direct evidence of the heterogeneity of cellular tension.

It is important to note that, compared to conventional FRET‐based MTPs, MIET‐TPM exhibits a lower SNR, particularly in podosome measurements. This limitation can lead to an underestimation of the measured height (Figure S10 and Note S6). Another limitation of MIET‐TFM is its sensitivity exclusively to the axial component of cellular forces. Since fluorescence lifetime changes report only on the *z*‐position of the fluorophore, lateral force components do not contribute to the signal and thus remain undetected. Consequently, variations in the measured height may reflect either changes in force magnitude or orientation–distinctions that cannot be resolved with the current approach.

In conclusion, we have demonstrated the feasibility of nanoscale axial imaging of tension probes actively unfolded by cellular machinery. The unique combination of MIET and MTP provides direct evidence of the nanoscale interactions between MTP and PM during adhesion formation across various adhesion structures. This approach can be extended to enable the simultaneous observation of MTPs alongside other force‐exerting molecules, such as actin, integrin, talin, and vinculin, offering detailed insights into the nanostructures and their interplays involved in force transmission. MIET‐TPM achieves super‐resolution in the axial direction; however, its lateral resolution remains constrained by the optical diffraction limit. Therefore, resolving axial forces with high lateral resolution remains an important objective for advancing force mapping at the nanoscale. Looking forward, we envision that our system could be integrated with single‐molecule localization techniques (e.g., DNA‐PAINT) to achieve 3D super‐resolution mapping of force events, enabling a deeper understanding of how precise local arrangements influence force‐signaling outcomes.

Because MIET also induces changes in fluorescence intensity, MIET‐TPM can be applied to all surface‐based and fluorescence intensity‐based force measurements, similar to conventional FRET‐based MTPs. This versatility makes it a promising technique for investigating various force‐involved receptor‐ligand pairs and their impacts on intracellular signaling. We anticipate that MIET‐TPM will become a standard methodology for bridging the fields of structural biology and mechanobiology, advancing our understanding of cellular force dynamics at the nanoscale.

## Conflict of Interests

The authors declare no conflict of interest.

## Supporting information

Supporting Information

## Data Availability

The data that support the findings of this study are available from the corresponding author upon reasonable request.
