# Supplementary material for: Super‐Resolution Axial Imaging for Quantifying Piconewton Traction Forces in Live Cells
Source: Angew Chem Int Ed Engl. 2025 Aug 18;64(41):e202506864. doi: 10.1002/anie.202506864 (PMC12501754; doi:10.1002/anie.202506864)
Supplement: Supplementary file 1 — Supporting Information [file ANIE-64-e202506864-s001.pdf]

Supporting Information

Super-Resolution Axial Imaging for Quantifying Piconewton Traction Forces in Live Cells

Dong-Xia Wang,[1,2,3] José Ignacio Gallea,[1] De-Ming Kong,[2,*] Jörg Enderlein,[1,3,*] Tao Chen[1,*]

[1]Third Institute of Physics – Biophysics, Georg August University, Friedrich-Hund-Platz 1, Göttingen, 37077, Germany.
[2]State Key Laboratory of Medicinal Chemical Biology, Tianjin Key Laboratory of Biosensing and Molecular Recognition, Frontiers Science Center for Cell Responses, Research Centre for Analytical Sciences, College of Chemistry, Nankai University, Tianjin, 300071, P. R. China.
[3]Department of Molecular Biology, Princeton University, Princeton, 08540, USA.
[4]Cluster of Excellence "Multiscale Bioimaging: from Molecular Machines to Networks of Excitable Cells" (MBExC), Universitätsmedizin Göttingen, Robert-Koch-Str. 40, Göttingen, 37075, Germany.
*Corresponding author:
kongdem@nankai.edu.cn;
jenderl@gwdg.de;
tao.chen@phys.uni-goettingen.de.

Abstract: Cell mechanics play a pivotal role in regulating numerous biological processes. While super-resolution microscopy enables the imaging of cellular forces in the lateral dimension with sub-10-nanometer resolution, achieving comparable resolution along the axial dimension remains a significant challenge. Here we introduce metal-induced energy transfer (MIET)-based tension probe microscopy (MIET-TPM), a technique for mapping cellular mechanical forces with nanometer precision in the axial direction. This approach combines the nanometer spatial resolution of MIET imaging with the piconewton sensitivity of DNA-hairpin-based molecular tension probes (MTPs), enabling the simultaneous observation of both the plasma membrane and force-exerting molecules in the axial dimension. Using MIET-TPM, we mapped axial integrin tension within focal adhesions and podosomes, alongside their corresponding plasma membrane height profiles, offering detailed insights into the nanoscale structures and mechanisms involved in force transmission. Notably, MIET-TPM can be implemented on any fluorescence microscopy setup without hardware modifications, making it a versatile and accessible tool that promises to become an integral part of future cellular mechanobiology analysis.

Experimental Procedures

MTP sensor synthesis and purification

All oligonucleotides (listed in Table S1) were ordered from Thermo Fisher, except for the ligand (DBCO-modified ssDNA FAM/Cy3), which was purchased from Biomers.net. A total of 5 nmol of DBCO-ligand and 30 nmol of Azide-cRGD (azide-modified cyclo [Arg-Gly-Asp-D-Phe-Lys(PEG-PEG)]) (Peptides International, cat. no. RGD-3759-PI) were conjugated in 1× PBS (pH 7.4) overnight at 15 °C, using a metal bath at 300 rpm in a total volume of 1 mL. The product was purified using a C18 reversed phase column (Thermo Fisher), with a gradient from buffer A (0.1 M TEAA triethylammonium acetate buffer) to buffer B (acetonitrile) over the course of 60 minutes (Figure S1). Peak fractions were collected and filtered through ultrafiltration centrifuge tubes with a molecular weight cutoff of 3.5 kDa. Successfully conjugated DNA ligands were stored at -20 °C until further use.

To synthesize MTP probes, three oligonucleotides at equal molar concentrations were mixed in 1× PBS (pH 7.4). The mixtures were annealed by heating to 95 °C, then cooled to 25 °C at a rate of 1 °C/min in a 0.2 mL thermowell tube. To chemically open MTPs, MTPs were hybridized with a 5× molar excess of complementary sequence.

Small unilamellar vesicle (SUV) preparation

To prepare SUVs, 100 µL of a 10 mg/mL solution of 1,2-dioleoyl-sn-glycero-3-phosphocholine (DOPC) lipids (Sigma-Aldrich, P6354) and 0.2 mol% biotinylated lipids (1,2-dioleoyl-sn-glycero-3-phosphoethanolamine-N-(biotinyl) (Biotinyl-Cap PE)) (Sigma-Aldrich, 870273P) in chloroform was vacuum-dried at 30 °C for 1 hour to remove the solvent. Subsequently, 500 µL of PBS buffer (pH 7.4) was added, and the mixture was incubated at 30 °C with shaking for 1 hour. The resulting lipid suspension was then extruded through a 50 nm polycarbonate filter (Whatman) for 15 cycles. These vesicle solutions should be used within 3 days and stored at 4 °C until use.

MIET calibration curves calculation

The geometry of a MIET experiment is illustrated in Figure S2. A fluorescent molecule is positioned at a distance z above a substrate, which consists of a 10-nm-thick gold layer and a 15-nm silica layer on a commercial glass coverslip. Fluorescence excitation and detection are conducted through this substrate from below. For calculating the MIET calibration curve (lifetime versus distance curve), the emitting molecule is treated as an ideal oscillating electric dipole, and its emitted electromagnetic field is mathematically described as a superposition of plane waves. The interaction of each plane wave with the planar substrate is calculated using the standard Fresnel theory, yielding the complete electromagnetic field of the emitter in the presence of the substrate. By integrating the Poynting vector two parallel planes enclosing the emitter, the total energy flux of the emitted field can be determined, enabling the calculation of the full emission rate of the dipole. By applying the same approach to two planes enclosing the MIET substrate (the metal layer), the fraction of energy absorbed by the substrate can be calculated. From these calculations, the emission rate $S(\theta, z)$ of an ideal electric dipole is found to be:

$$S(\theta, z_0) = S_{\perp} cos^2\ \theta + S_{\parallel} sin^2\ \theta \quad (1)$$

where θ represents the angle between the dipole's axis and the vertical direction (normal to the surface), and the functions $S_{\perp}$and $S_{\parallel}$ depend solely on the orientation θ.

Additionally, real fluorophores exhibit nonradiative transitions from the excited to the ground state, which determines the quantum yield φ of the fluorophore:

$$\varphi = \frac{k_r}{k_{nr} + k_r} \quad (2)$$

Here, k_r is the radiative transition rate, and k_{nr} is the nonradiative transition rate. When comparing the fluorescence lifetime of a free molecule far from the substrate to that of a molecule at a distance z above the substrate, we get:

$$\frac{\tau_0}{\tau_{(\theta,z)}} = \frac{k_r(\theta,z) + k_{nr}}{k_{r,0} + k_{nr}} = \frac{S(\theta,z)}{S_0}\varphi + 1 - \varphi \quad (3)$$

where S_0 represents the free-space emission power of an ideal electric dipole emitter and is given by $S_0 = cnk_0{}^4 p^2/3$, with c being the speed of light, k_0 the wavevector in vacuum, n the refractive index of water, and p the amplitude of the dipole moment vector. Finally, the fluorescence lifetime $\tau_{(\theta,z)}$ as a function of distance z is fitted using Equation3, with the angle θ, the free-space lifetime τ_0, and the quantum yield value φ as fitting parameters. The free-space lifetimes for all fluorophores were measured using samples on glass surfaces (Figure S3). Additional details regarding the determination of fluorescence lifetimes, quantum yields, and fluorophore orientations are provided in Supporting Note 1. All fitting parameters are listed in Supporting Table 2, and the calculated MIET curves are shown in Figure S4.

Substrate preparation

A layer-by-layer electron-beam evaporation process was used to deposit a 2~nm titanium layer, followed by a 10~nm gold layer, another 1~nm titanium layer, and finally a 15~nm SiO_2 layer on the surface of a glass coverslip. The deposition rate was kept at the slowest rate (1 $Ås^{-1}$) to ensure maximal homogeneity. The spacer thickness was continuously monitored during evaporation using an oscillating quartz unit. This gold-covered substrate is referred to as the MIET substrate.

To prepare the SLB on the MIET substrate, the MIET coverslip was first activated for 30 seconds with low-intensity plasma from a plasma cleaner (Harrick Plasma, New York, USA). After activation, a droplet of the SUV solution was placed onto the substrate and incubated for 1 hour to promote the formation of a continuous bilayer with minimal imperfections. The substrate was then thoroughly washed with 1× PBS buffer. Next, the substrate was incubated with Neutravidin (Thermo Fisher Scientific, cat. no. 31000) at a final concentration of 0.5 mg/mL for 20 minutes. After another wash with 1× PBS, the substrate was incubated for 60 minutes with 200 nM of biotin-labeled MTP and subsequently washed again with 1× PBS.

For the hard substrate, after the MIET coverslip was activated, biotin-labeled BSA (Sigma-Aldrich, A8549) at 500 µg/mL concentration was added and incubated for 20 min. After washing with water, neutravidin solution was added directly without the addition of SUVs. The remaining steps were identical to those used for preparing the soft substrate.

Cell culture and imaging

Cells were cultured in Dulbecco's Modified Eagle Medium (DMEM) supplemented with 10% (v/v) fetal bovine serum and 1% antibiotics (penicillin-streptomycin-amphotericin B) until they reached approximately 75% confluency. The cells were then detached from culture flasks using a 0.25% (wt/vol) trypsin solution (Corning). The prepared cells were seeded onto MTP-functionalized surfaces at a density of 20,000 cells/cm^2. Imaging of the cells was conducted between 20 and 120 minutes post-plating.

Immunostaining

NIH 3T3 fibroblasts were seeded onto SLBs functionalized with 4.7 pN MTPs and allowed to spread for 90 minutes. Cells were then fixed with 4% formaldehyde for 20 minutes at room temperature, permeabilized with 0.1% Triton X-100 for 10 minutes, and blocked with 1% BSA for 30 minutes. Primary staining was performed using anti-vinculin antibody [EPR8185] (1:200, ab129002, Abcam) for 1 hour at room temperature. This was followed by incubation with a secondary antibody (Goat Anti-Rabbit IgG H&L, Alexa Fluor® 488, 1:200, ab150077, Abcam) and Alexa Fluor® 647-conjugated phalloidin (1:1000, #8940, Cell Signaling Technology) to label F-actin.

Results and Discussion

Note 1. Determination of fluorescence lifetimes, quantum yields, and orientations of fluorophores for calculating the MIET calibration curves.

To determine the fitting parameters for MIET calculation, the free-space lifetime τ_0 was measured using the samples (Cy3-MTP, FAM-MTP, and CellMask Deep Red (CMDR)) on a bare glass surface (see Figure S3). For Alexa Fluor® 488 conjugated antibodies, we observed that the fluorescence lifetime exhibited slight variations depending on incubation time and concentration. To account for this, we employed a half-gold/half-glass coverslip configuration to determine the free-space lifetime under identical experimental conditions within the same chamber for each measurement. The fluorescence quantum yields (∅) of the fluorophores were calculated using a relative method by comparing the intensity of standard fluorescence with that of the unknown samples (Cy3-MTP and FAM-MTP).[1] For this, we used fluorescein (∅ = 0.925 in 0.1 M NaOH)[2] as the references for the FAM-MTP and tetramethylrhodamine (TMR, ∅ = 0.68 in methanol)[3] as reference for the Cy3-MTP.

It is important to note that the lifetimes and quantum yields of fluorophores are sensitive to the DNA structures and cell type,[3–5] we strongly recommend that anyone calculating the MIET curves measure the free-space lifetime and quantum yields specific to their own systems prior to data quantification.

The orientation of fluorophore was taken from the literature. The 6-carboxyfluorescein (FAM) fluorophore, a fluorescein-based dye, has been reported to exhibit a random orientation within DNA structures.[6] In contrast, the cyanine-based dye Cy3 would stack against the nucleobases of the duplex perpendicular to its long axis.[4,6] Previous reports have revealed tilt angles of the DNA duplex from the normal to be 21° ± 2° for podosomes and 40° ± 2° for focal adhesions. Therefore, the tilt angles of Cy3 were 69° and 50° from vertical for those two structures, respectively, and these values were used in calculating the MIET curves of Cy3 fluorophore. For the plasma membrane (PM) staining dye CMDR, our group has already determined that it has a random orientation.[7,8] All parameters used for calculating the MIET curves are listed in Table S2 and the calculated MIET curves are shown in Figure S4.

Note 2. No FRET between Cy3-MTP and Cellmask Deep Red on the cell.

The overlap between the fluorescence spectrum of Cy3 and absorption spectrum of CMDR would potentially induce FRET between these two dyes if the distance between them is less than 10 nm. However, in our systems, this effect is not present because the distance between Cy3 and CMDR is sufficiently larger. It has been reported that the extracellular domain of integrin adopts an upright conformation upon activation[9] and stands approximately 20 nm above the membrane surface[10]. Additionally, the DNA duplex between the dye and RGD is approximately 8.2 nm in length. Even if the DNA duplex has some tile, the substantial distance between the dye and the membrane ensures that FRET not occur in our system.

To experimentally confirm our estimation, we compared the podosome force maps using Cy3- MTPs with CMDR and without CMDR for one same cell. After collecting the Cy3 signal from one cell, we added CMDR to the observation chamber, carefully washed the chamber, and then collected the Cy3 signal at the same position. As shown in Figure S5, the height results are nearly identical for both measurements.

Note 3. Lifetime determination based on the maximum likelihood estimation

The fluorescence lifetime value for each pixel was determined by fitting the tail (starting 0.3 ns after maximum) of the TCSPC curves using a maximum likelihood estimation (MLE) method. MLE estimates decay times by minimizing the likelihood function.[11,12] It has been demonstrated that MLE is a robust method for precisely estimating fluorescence lifetime, even for decays with total counts of less than 200. Specifically, MLE provides a 20% standard error even at 20 total counts, less than 10% error at 200 total counts, and approximately 2% error when total counts > 1000.[11–13] In our measurement, the minimum counts per pixel were approximately 400 for FA measurement, ~800 for podosome measurement, and ~ 1500 for PM measurement (See Figure S9).

To evaluate the accuracy of our fitting method at different counts, we measured a standard fluorophore sample with a well-known fluorescence lifetime (atto 655 in PBS, τ = 1.80 ns).[14] We then divided the total recorded photons into bunches of different photon counts (N = 200, 400, 1000, and 7000, Figure S8). The corresponding TCSPC curves were fitted using our MLE method, and their deviations were compared to the standard lifetime. As shown in Figure S8, the fitted lifetime value at 400 total counts was approximately 4% lower than the expected value, with a standard error of around 8% standard error (SD/mean).

To further estimate the height error resulting from the lifetime fitting at different counts, we constructed height maps at varying photon numbers per pixel by binning frames for a sample of FAM-MTP on SLB supported by a MIET substrate. This sample was homogeneous due to fluorophore diffusion. As shown in Figure S9, at very low counts (77 counts), the calculated mean height is 16.1 ± 2.4 nm, which is only 1 nm lower than the value calculated with 1600 counts (17.1 ± 1 nm). The mean height calculated with 320 counts is 16.5 ± 1.6 nm, demonstrating the height error in our calculation (with a minimum 400 counts used) is less than 2 nm.

Note 4. Calculation of the fluorophore's heights in MTP on the surface of solid MIET substrate and SLB.

The orientations of Cy3 fluorophores in the MTP attached to integrins have been determined in previous reports.[6,15] However, their orientations on solid substrate and SLB without cells are still unknown, making it necessary to determine the fluorophores' height when the MTP is in a closed state on these substrates. To determine the fluorophore's height, we used another dye, FAM, to label the same position on the MTP. Unlike Cy3, FAM does not stack with the DNA duplex and assumes a random orientation.[6] As shown in Figure

S7, the MTPs on both solid substrate and SLB exhibit very homogeneous distributions. The mean heights of the FAM fluorophore in a 4.7 pN MTP are 17.6 ± 1.7 nm for the solid substrate and 17.1 ± 1.0 nm for the SLB.

Additionally, we did not use FAM as the main fluorophore for our cellular measurements because we found that FAM is much more prone to photobleaching and is significantly dimmer than the Cy3 tension probe, which limits imaging quality.

Note 5. Limitations of MIET on localizing FA proteins

It should be noted that focal adhesion (FA) proteins such as vinculin are located inside the cell and typically reside above the plasma membrane—outside the optimal axial working range of MIET (approximately 5–70 nm for most fluorophores). In this higher axial region (>70 nm), even small uncertainties in lifetime measurements can lead to significant errors in height estimation. For example, as shown in Figure S13 (MIET curve for Alexa Fluor® 488–labeled antibodies), a 2% uncertainty in fluorescence lifetime translates to an error of approximately 12 nm in axial distance. More accurate co-localization of multiple FA proteins using MIET is possible for cell types with thinner ECM between the basal surface and the MIET substrate.[7,16] However, in the NIH 3T3 cells used in this study, the larger vertical spacing between the target proteins and the MIET substrate limits the precision of MIET. Therefore, it remains challenging to distinguish the axial positions of different FA proteins with high accuracy (<5 nm) in the current experimental context.

Note 6. Limitations of MIET on localizing the axial tension force of integrin

1) Difficult to determine the percentage of open probe

The open percent of the DNA hairpin is a critical parameter for evaluating integrin force.[6,15,17,18] However, in our system, determining open percentage is challenging due to the tilt angles of DNA structures on both solid substrates and SLBs, and because MIET can only resolve the vertical position. Consequently, we cannot generate a calibration curve for calculating the open percent from either fluorescence lifetime or intensity.

2) Ensemble averaging

The measurement of lifetime (or height) is influenced by ensemble averaging of the fluorescence of many fluorophores within the confocal focus. The observed signal is a spatial average over the size of the excitation focus (lateral region of approximately 300 nm), resulting in an averaged outcome for both open and closed DNA hairpins. This ensemble averaging tends to underestimate the lifetime value. However, unfolding probes positioned higher emit more photons, which reduces the underestimation of lifetime. We further analyzed the signal-to-noise ratio (SNR) to evaluate this effect on height mapping for different components.

3) Signal to Noise Ratio

The SNR analysis for all components (FA-MTP, Podosome-MTP, and PM) is detailed in Figure S10: (i) For the FA-MTP, the mean background count is 41 counts. In the height map analysis, only pixels with counts greater than 400 (representing regions of the FA) were included. Therefore, the SNR for the FA-MTP is greater than 10, allowing us to ignore the underestimation effect of the folding probes for FA height mapping. (ii) For the podosome-MTP, the mean background count number is 650, and the mean count in the podosome region (count > 1000) is only 1250, resulting in an SNR of approximately 2. This low SNR leads to a larger error in the lifetime calculation from the unfolding MTP. Since half of photons in the lifetime fitting come from folding MTP s, the estimated actual lifetime of the podosome region is affected. The low SNR may be due to fluorophore diffusion, minimal height increase, and a low open percentage. The fast diffusion of Cy3-MTP compensates for photobleached probe, leading to photon accumulation in the background when scanning the same area repeatedly. (iii) For the PM, the SNR is consistently greater than 10 because only the plasma membrane is labelled by the CMDR (Figure S10).

4) Fluorophore orientation

Determining the orientation of the fluorophore is crucial for calculating the MIET calibration curve. However, the presence of cyanine dyes can complicate this calculation due to their tendency to stack against the DNA duplex. While previous studies have provided insight into the average tilt angle of the fluorophore,[6,15,18] determining the orientation for focal adhesions (FAs) presents additional challenges. The tilt angle of FAs varies depending on their position within the cell: FAs exert more vertical forces at the cell center, with a median DNA tilt angle of 30°, whereas they become increasingly lateral near the cell periphery, with a median DNA tilt angle of 41°. Given that our analysis focuses solely on FAs at the cell periphery, we used a DNA tilt angle of 40° for our MIET calculation. Importantly, this 10° difference is expected to introduce only a negligible ~2 nm height error during the conversion process.

Table S1: Oligonucleotide sequences used in this work are listed above.

Name	Sequence (5' to 3')
Ligand (ssDNA FAM)	DBCO-TTT GCT GGG CTA CGT GGC GCT CTT-FAM
Ligand (ssDNA CY3)	DBCO-TTT GCT GGG CTA CGT GGC GCT CTT-Cy3
Anchor	CGC ATC TGT GCG GTA TTT CAC TTT-biotin
Hairpin-4.7 pN	GTG AAA TAC CGC ACA GAT GCG TTT GTA TAA ATG TTT TTT TCA TTT ATA CTT TAA GAG CGC CAC GTA GCC CAG C
Hairpin-19 pN	GTG AAA TAC CGC ACA GAT GCG CGC CGC GGG CCG GCG CGC GGT TTT CCG CGC GCC GGC CCG CGG CGA AGA GCG CCA CGT AGC CCA GC
cDNA-4.7 pN	AAA GTA TAA ATG AAA AAA ACA TTT ATA CAA A
cDNA-19 pN	CG CCG CGG GCC GGC GCG CGG AAA ACC GCG CGC CGG CCC GCG GCG

Table S2 Parameters used for calculating MIET calibration curves.

	Quantum yield, ∅	Free-space lifetime τ_0 (ns)	Orientation
FAM	0.53	3.3	Random
Cy3	0.42	1.8	69° and 50°
CellMask Deep Red (CMDR)	0.3	1.62	Random
Goat Anti-Rabbit IgG H&L, Alexa Fluor® 488	0.67	3.0 - 3.5 ns	Random

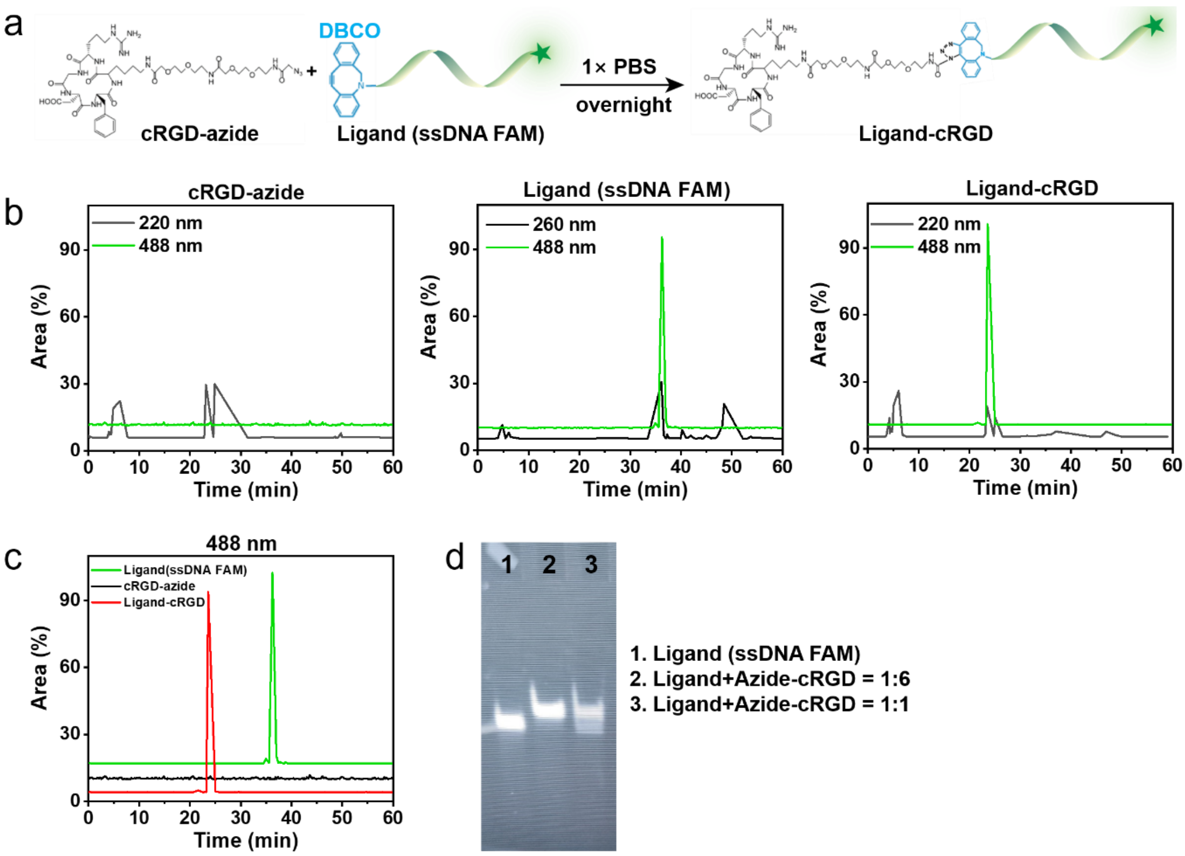

Figure S1. HPLC and PAGE characterization of modified probes. (a) Schematic showing the coupling between cRGD-azide and ligand (ssDNA-FAM) to form ligand-cRGD. (b) HPLC spectra of all the starting material (cRGD and oligonucleotides) as well as the products generated in this work. (c) HPLC spectra of all oligonucleotide products under 488 nm excitation. Solvent program: 0.5 mL/min flow rate; Solvent A: 0.1 M TEAA triethylammonium acetate buffer, Solvent B: acetonitrile. Starting condition: 0-10 min 100% A; 10-40 min 0-100% gradient B. (d) 10% polyacrylamide gel electrophoresis (PAGE) characterization of modified probes. When the ratio of ligand to cRGD is 1 : 6, the reaction efficiency reaches 100%, indicating that all oligonucleotides are connected to cRGD.

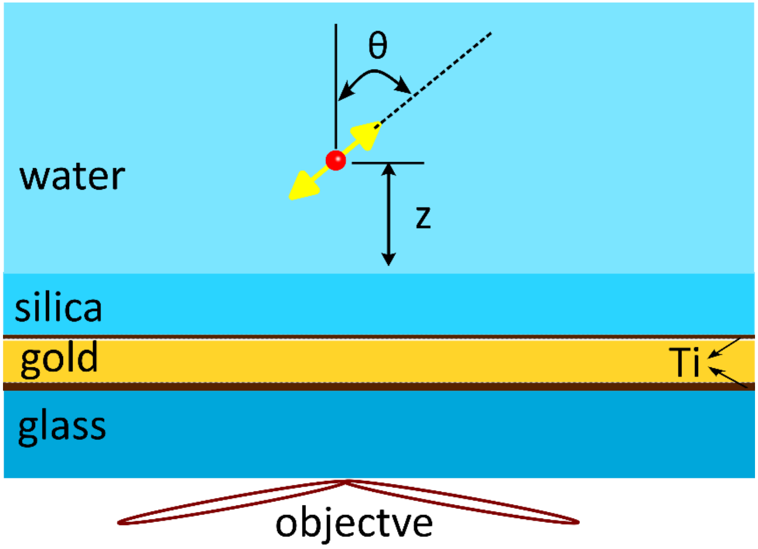

Figure S2. Geometry of MIET setup. A fluorophore is positioned above a MIET substrate comprising multiple layers: 15 nm silica, 1 nm Ti, 10 nm Au, and 2 nm Ti on a commercial coverslip. Fluorescence detection and excitation are conducted using a high numerical aperture objective (Apo N, 100× oil, 1.49 NA, Olympus Europe, Hamburg, Germany) from the glass side. The fluorophore is described as an electric dipole emitter, placing at a distance z from the silica surface and its orientation is the angle θ between its dipole axis and the optical axis.

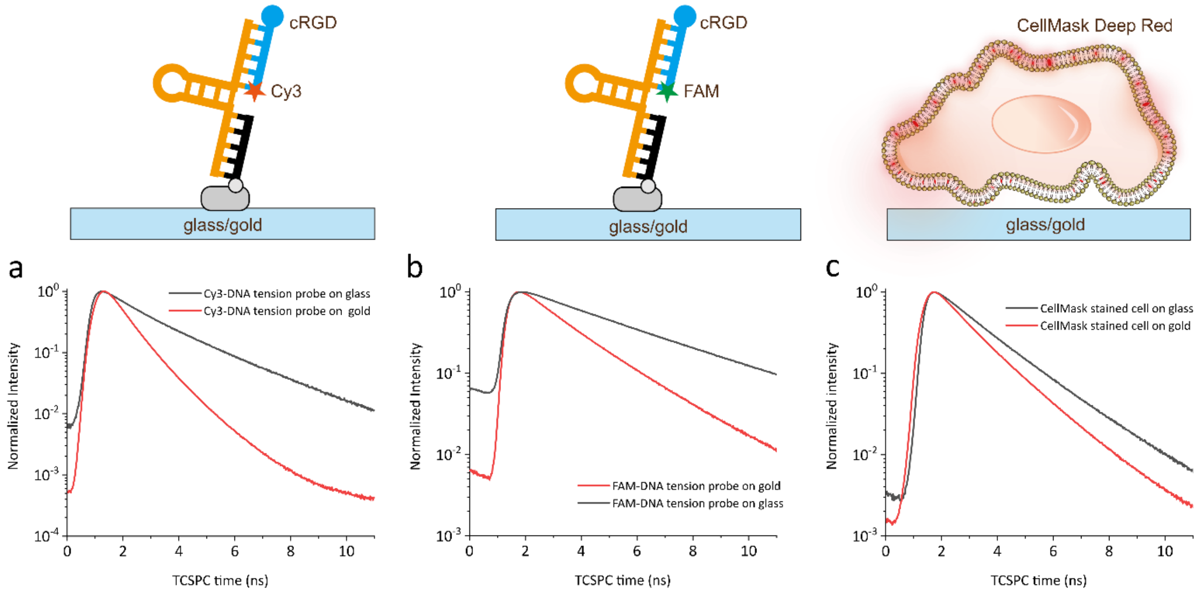

Figure S3. Fluorescence lifetime decay curves. (a) Fluorescence lifetime decay curves for Cy3-MTP measured on glass surface and gold surface. (b) Fluorescence lifetime decay curves for the FAM-MTP measured on glass surface and gold surface. (c) Fluorescence lifetime decay curves for CMDR-stained cells measured on glass surface and gold surface.

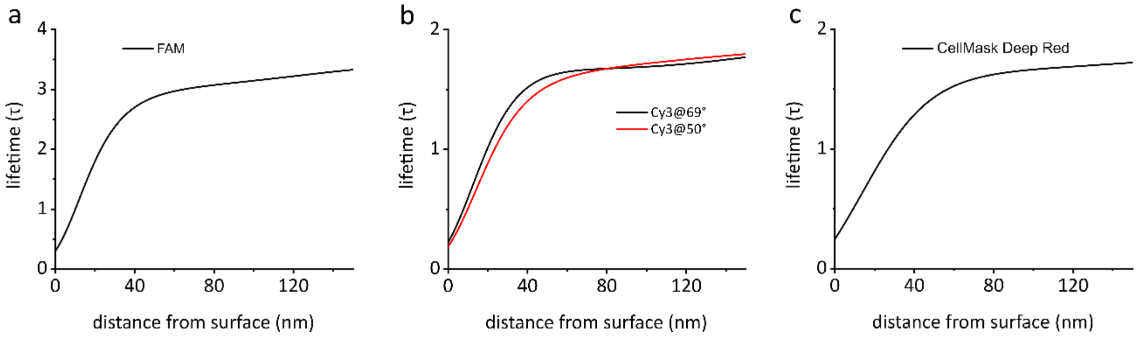

Figure S4. Calculated MIET calibration curves. (a) Calculated MIET calibration curve for FAM. (B) Calculated MIET calibration curve for Cy3 with two different orientation angles. (c) Calculated MIET calibration curve for CMDR. The optical parameters of the fluorophores used for these calculations are listed in Table S2. All calculations were performed for a MIET substrate consisting of 15-nm silica, 1 nm Ti, 10 nm Au, and 2 nm Ti on glass coverslip.

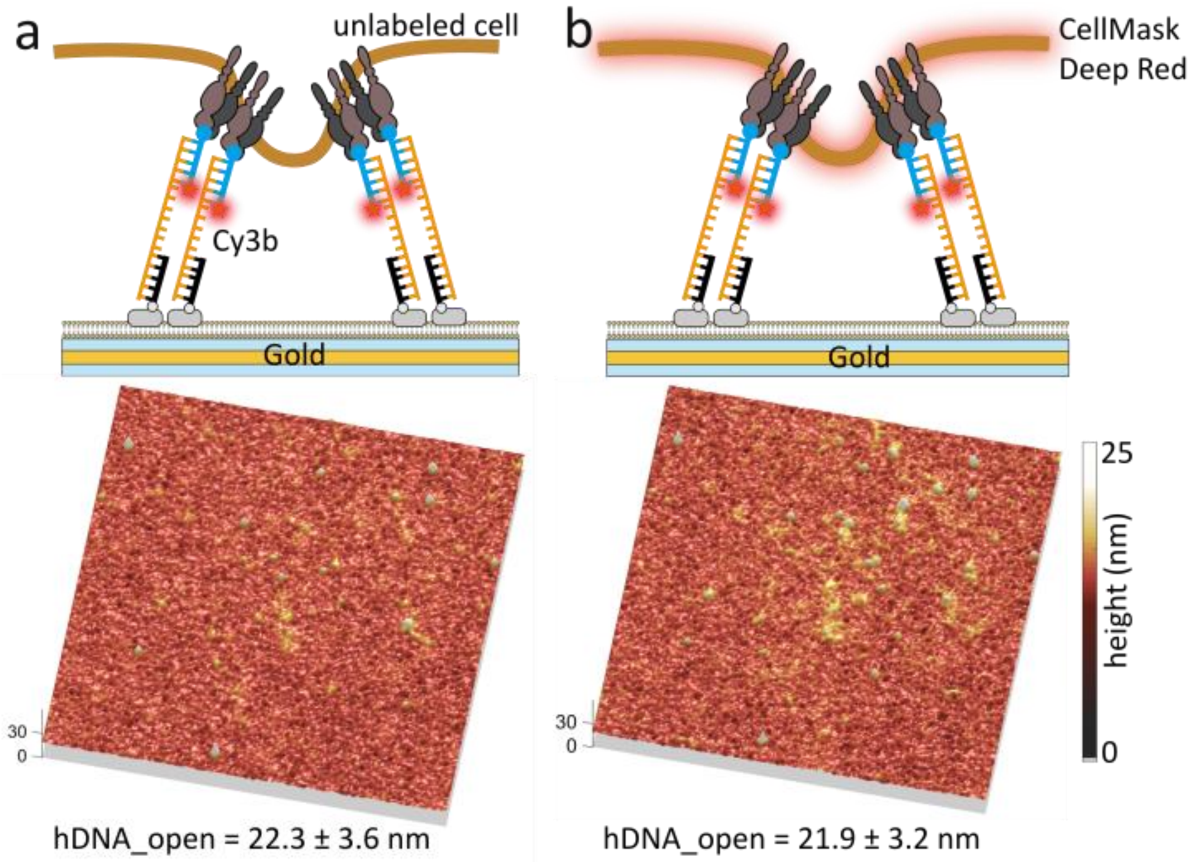

Figure S5. Confirmation of no FRET between Cy3-MTP and CMDR-PM. (a) Cy3-MTP height profile for the unlabeled cell on Cy3-MTP-SLB MIET substrate. (b) Cy3-MTP height profile for the CMDR-labeled Cell on Cy3-MTP-SLB MIET substrate.

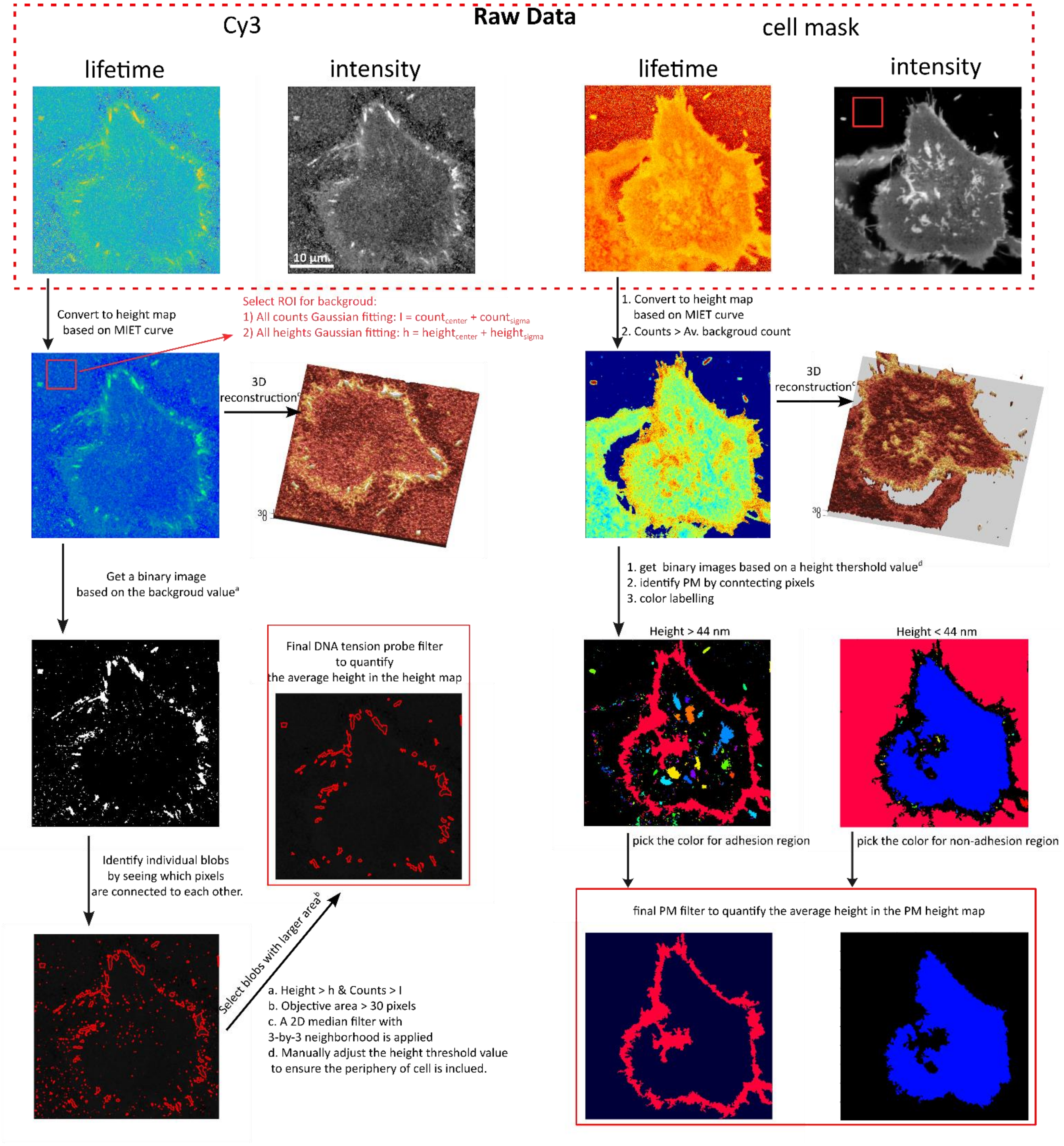

Figure S6: MIET analysis flowchart. The *.ptu raw data file is processed using custom Matlab routines to generate intensity and lifetime images for each color channel. The lifetime image is then converted into height map using the lifetime-to-height calibration curve (MIET curve). To extract heights for different components (opened DNA probes, PM under adhesion, and PM under non-adhesion), segmentation and feature extraction are performed on the height maps using the Image Processing Toolbox (Image Segmentation) in Matlab. For identifying focal adhesion, the height image is first converted into a binary image based on a height threshold. Subsequently, each focal adhesion is identified by connecting the pixel that are contiguous. Blobs with small areas are excluded, resulting in an image filter specific to focal adhesions. This filter is then applied to the height map to quantify the average height of the DNA probes in the focal adhesion area. The analysis for the PM follows a similar approach, with additional steps to label connected areas with different colors and select the edge area for the adhesion region and the central area for the non-adhesion region. Finally, filters for the PM height map are obtained to quantify the average height values.

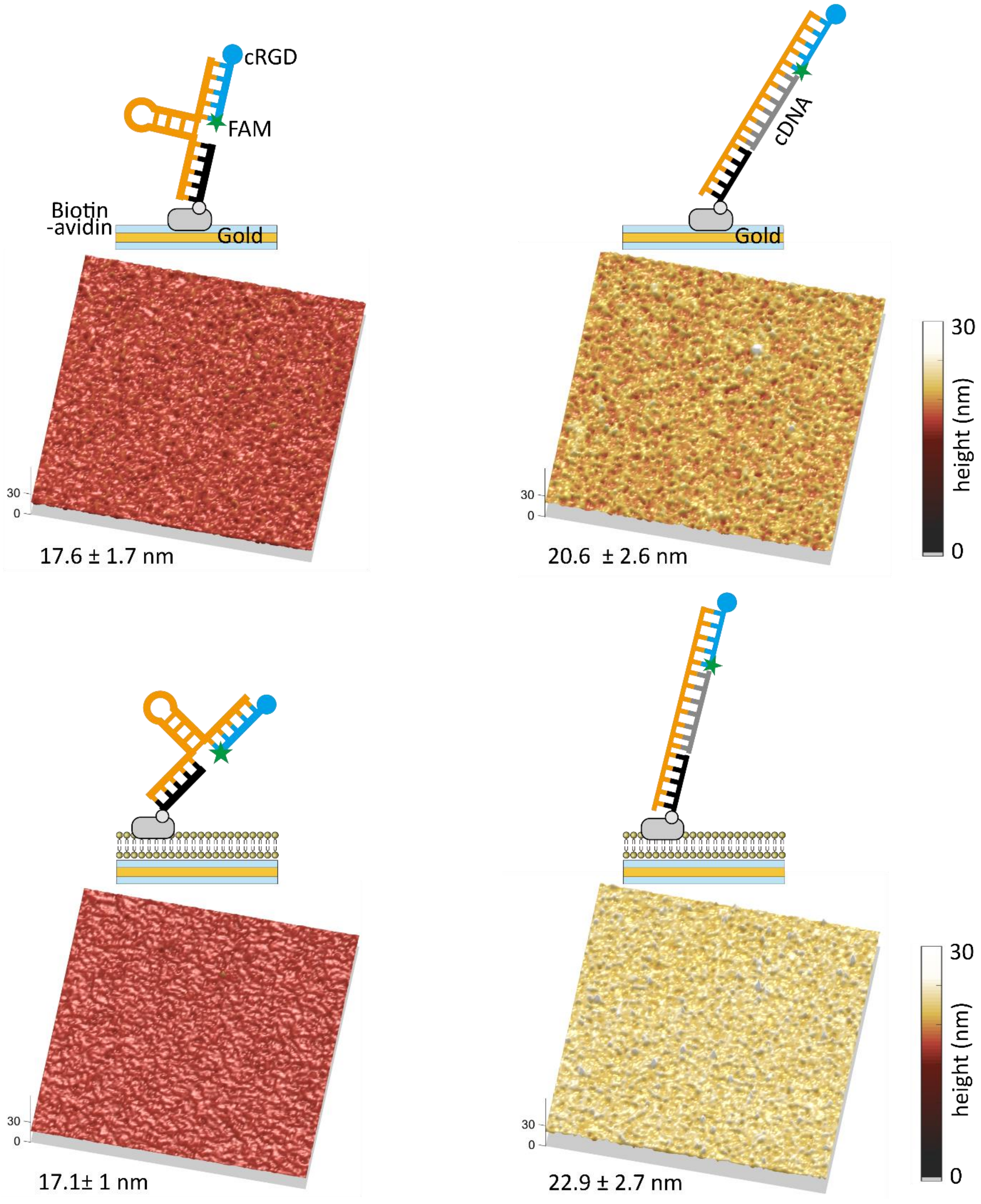

Figure S7. The height changes when the FAM-DNA-tension probes are unfolded with complementary strand. Schemes and corresponding height maps showing folded (left) and unfolded (right) FAM-MTP on solid (up) MITE surface and SLB-MIET surface (bottom). Compared to the height of the MTP caused by force, the reduced height observed in the cDNA-unfolded state likely results from probe tilt and partial surface adherence under zero force.

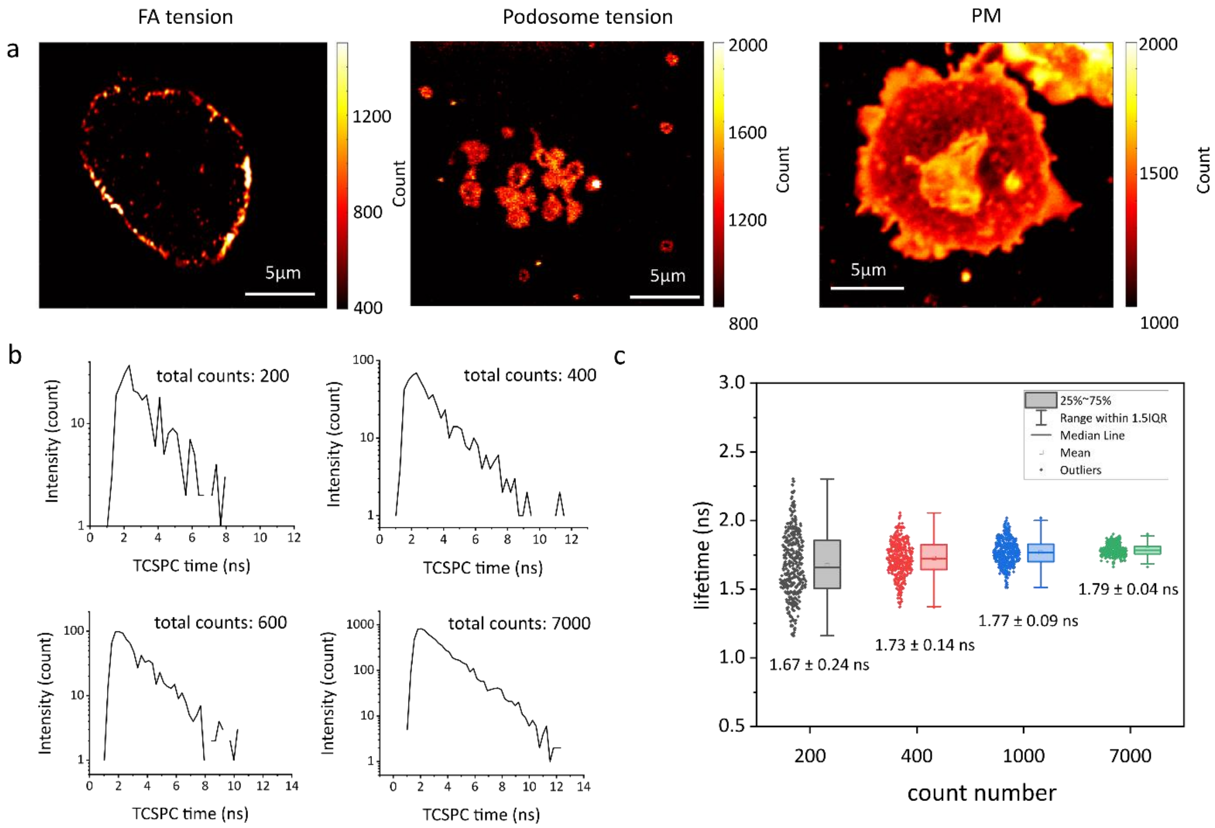

Figure S8: Fluorescence lifetime calculation. (a) Representative fluorescence intensity images of FA tension, podosome tension and PM. (b) Representative TCSPC curves constructed with different photon counts for the fluorophore Atto 655 in PBS solution. (c) Fitted lifetime distributions of TCSPC curves constructed with different counts for the fluorophore Atto 655. The elements of the box plots are explained in the figure. All values are expressed as the mean ± SD. For each dataset, 400 TCSPC curves are used for fitting.

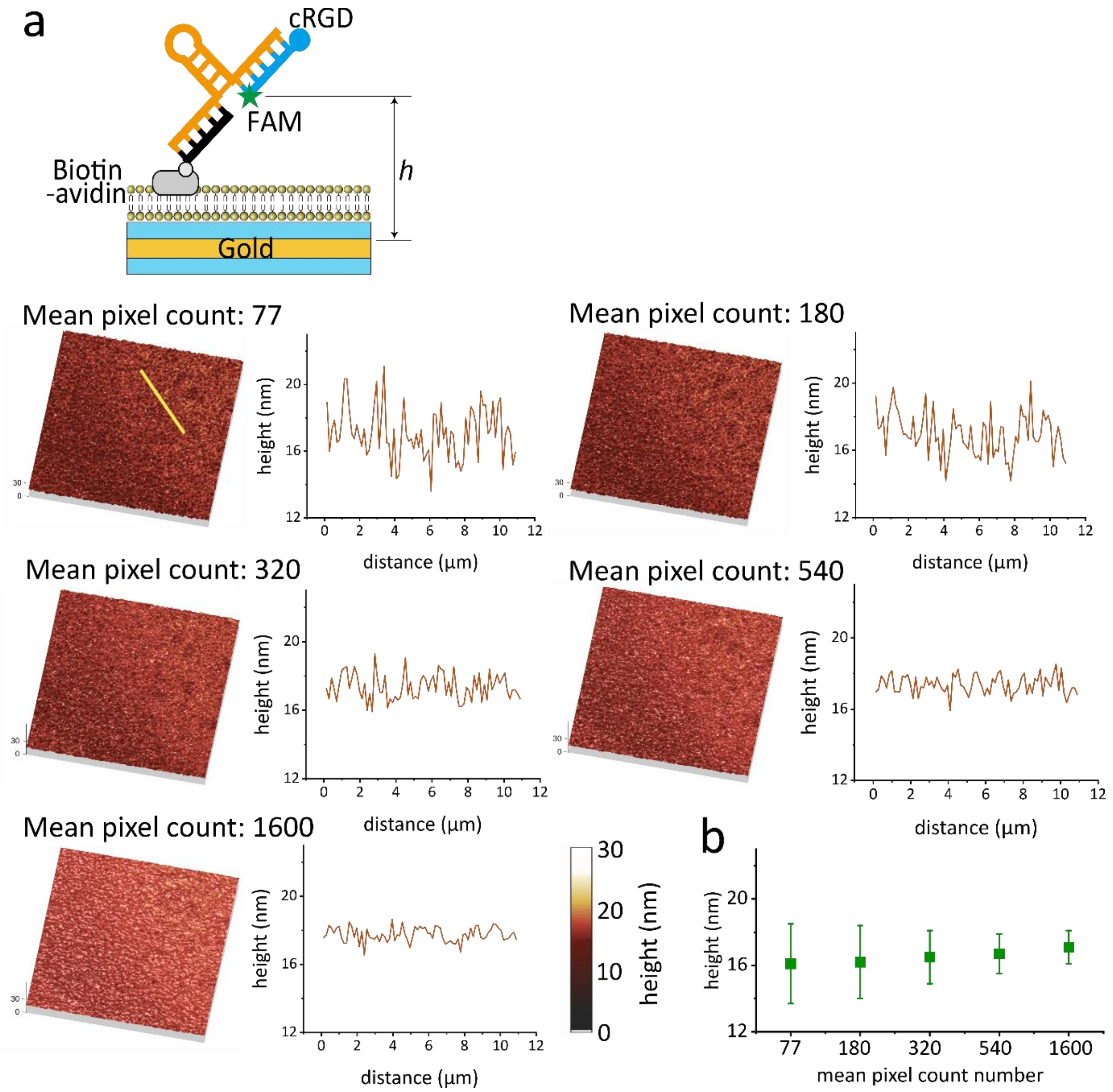

Figure S9: Estimation of height error based on the count numbers. (a) The left panel displays reconstructed height maps calculated using different counts numbers, while the right panel shows the corresponding cross-section height profiles for a line marked in the first image. The color and region of color bars for all height maps are consistent. (b) The mean height values for all pixels (200 × 200) in panel (a) are depicted, with error bars representing the standard deviation.

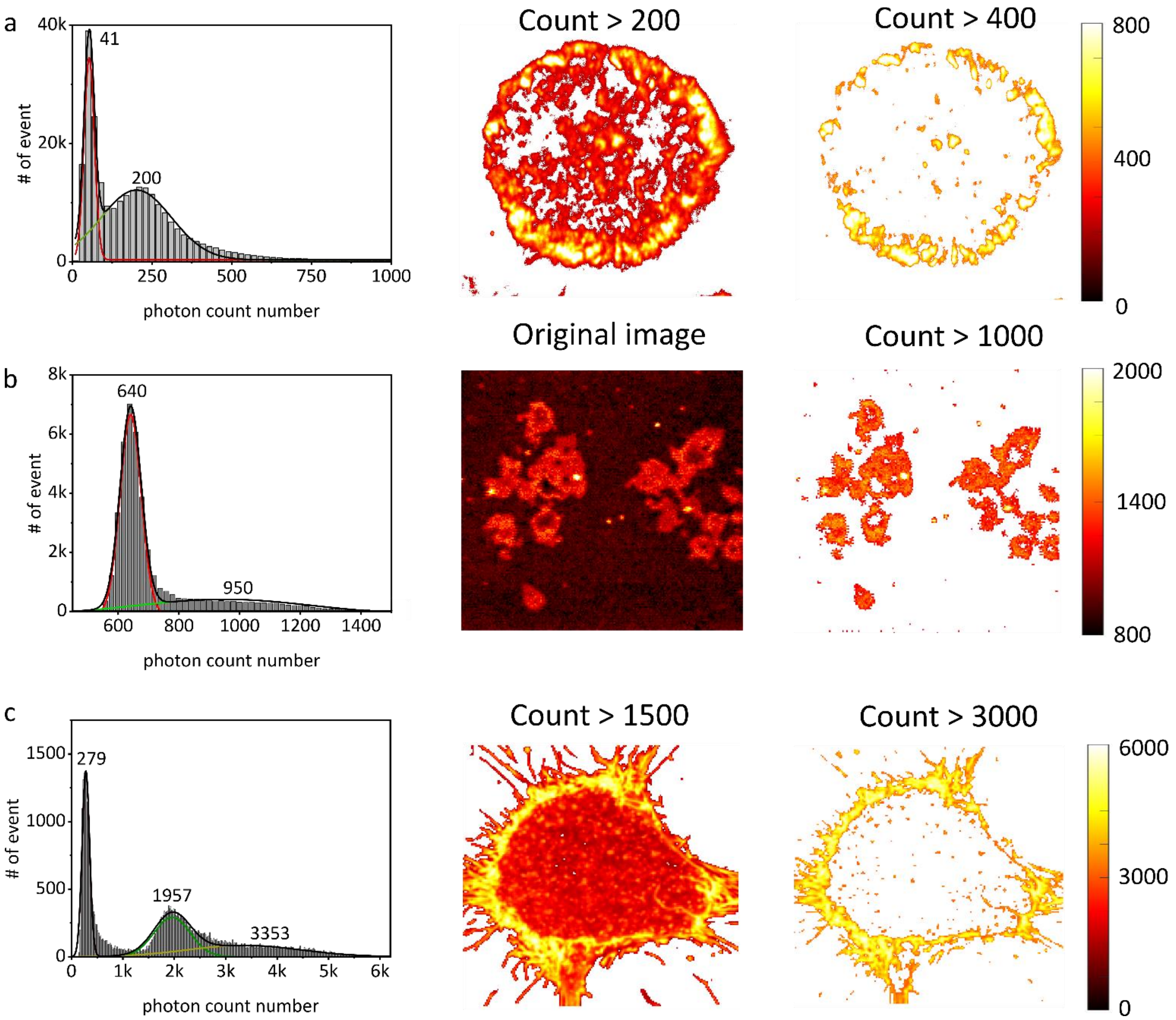

Figure S10. Signal-to-noise ratio analysis. Histogram analysis of the average photon counts per pixel, along with the corresponding photon count images filtered at different count threshold for (a) FA-MTP measurement, (b) podosome-MTP measurement, and (c) PM measurement.

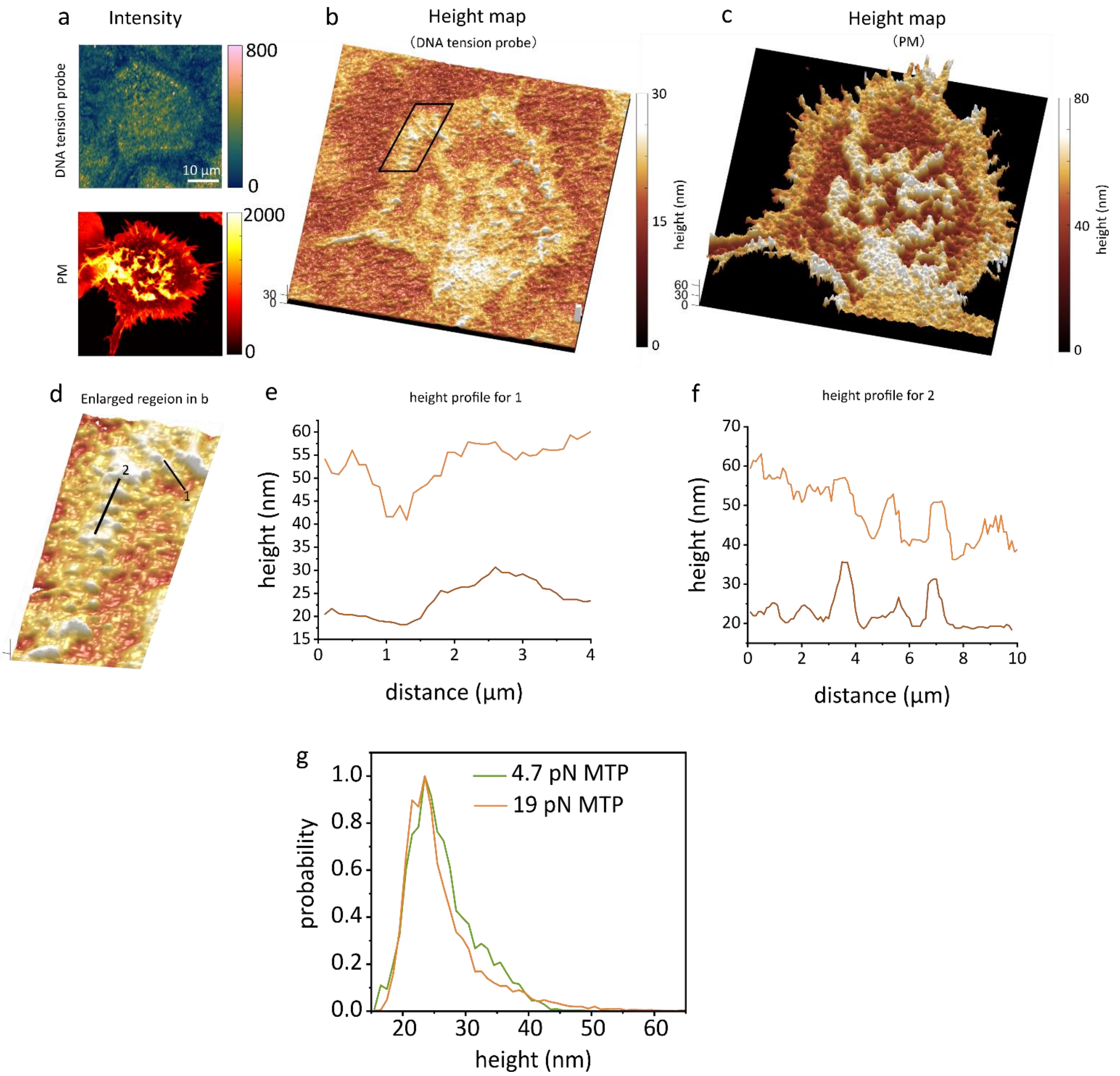

Figure S11. MIET measurement on 19 pN Cy3-MTP on solid substrate. (a) Fluorescence intensity images for both Cy3-MTPs and PM. (b, c) The 3D-reconstructed height map for Cy3-MTP and PM. (d) An enlarged region marked in panel b. (e, f) Height profiles of the Cy3-MTP and PM for line 1 and line 2. (g) Histogram of MTP heights for 4.7 pN and 19 pN MTP, respectively.

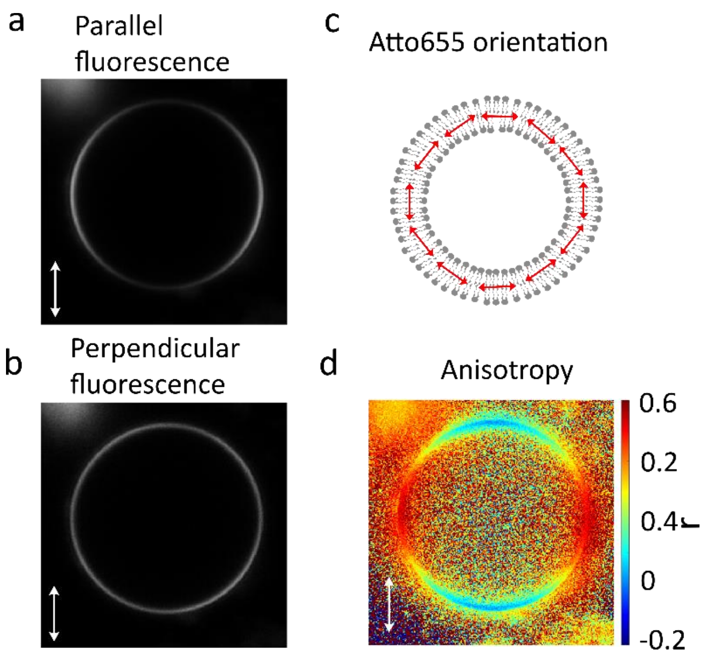

Figure S12. Anisotropy measurement. (a) Representative fluorescence intensity image of Atto655-DPPE labelled GUV. The fluorescence was excited using polarized light, and the emission was collected with parallel orientation. (b) Similar as a but the emission was collected with the perpendicular orientation to the excitation polarization. (c) The orientation of Atto655-DPPE in GUV, known to align parallel to the membrane. (d) Fluorescence anisotropy image. Fluorescence anisotropy was calculated as $r = (I_{\parallel} - I_{\perp})/(I_{\parallel} + 2I_{\perp})$, where $I_{\parallel}$ and $I_{\perp}$ are the fluorescence intensities with polarization parallel and perpendicular to the excitation polarization. The results clearly demonstrate that the anisotropy image reveals the orientation of fluorophore with respect to the excitation polarization.

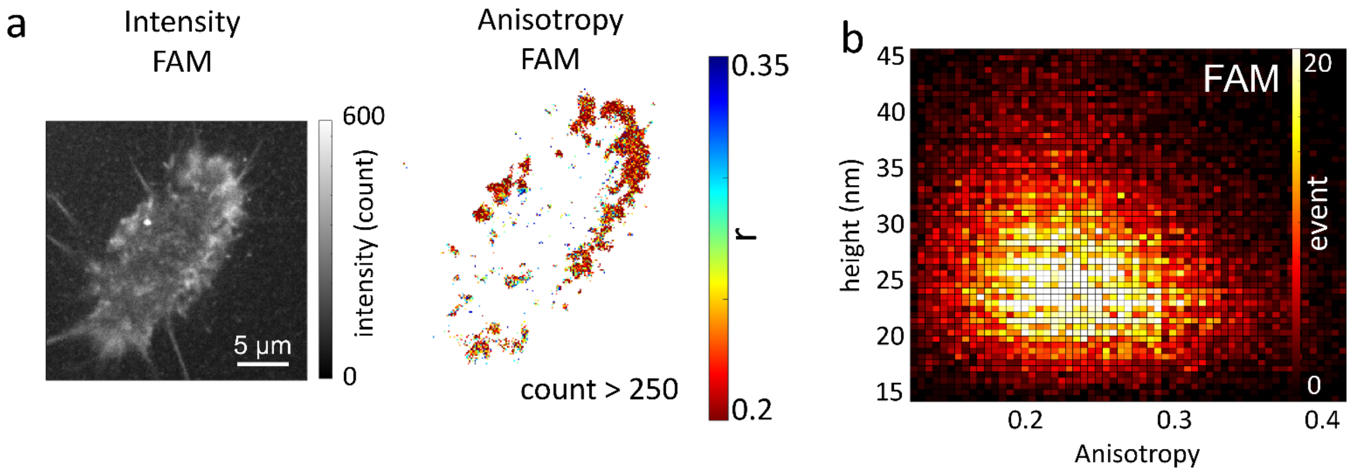

Figure S13. Anisotropy measurements of FAM-labeld MTPs. (a) Fluorescence intensity image and calculated anisotropy image of a cell on 4.7 pN FAM-MTP-modified MIET substrate. (b) 2D histogram depicting the relationship between the height and anisotropy for FAM-modified MTP.

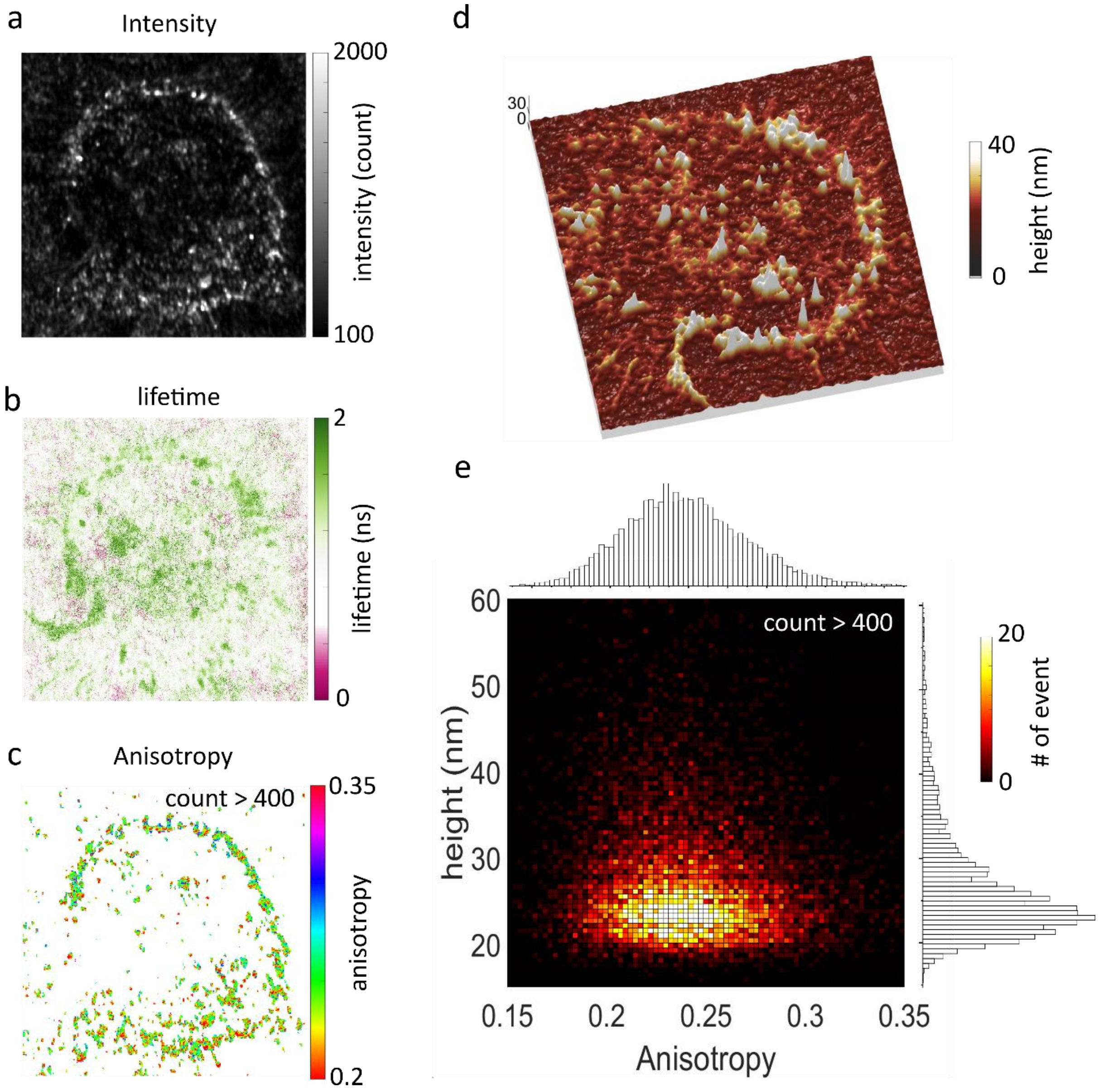

Figure S14. Anisotropy measurement on 19 pN Cy3-MTP on solid MIET substrate. (a) Fluorescence intensity image of 19 pN Cy3-MTP for a Cos7 cell. (b) The corresponding lifetime image, and (c) anisotropy image. (d) 3D-reconstructed height image. (e) 2D-histograms analysis of the anisotropy versus height. The histogram is constructed by taking all the pixels values from anisotropy image and height image over the count greater than 400.

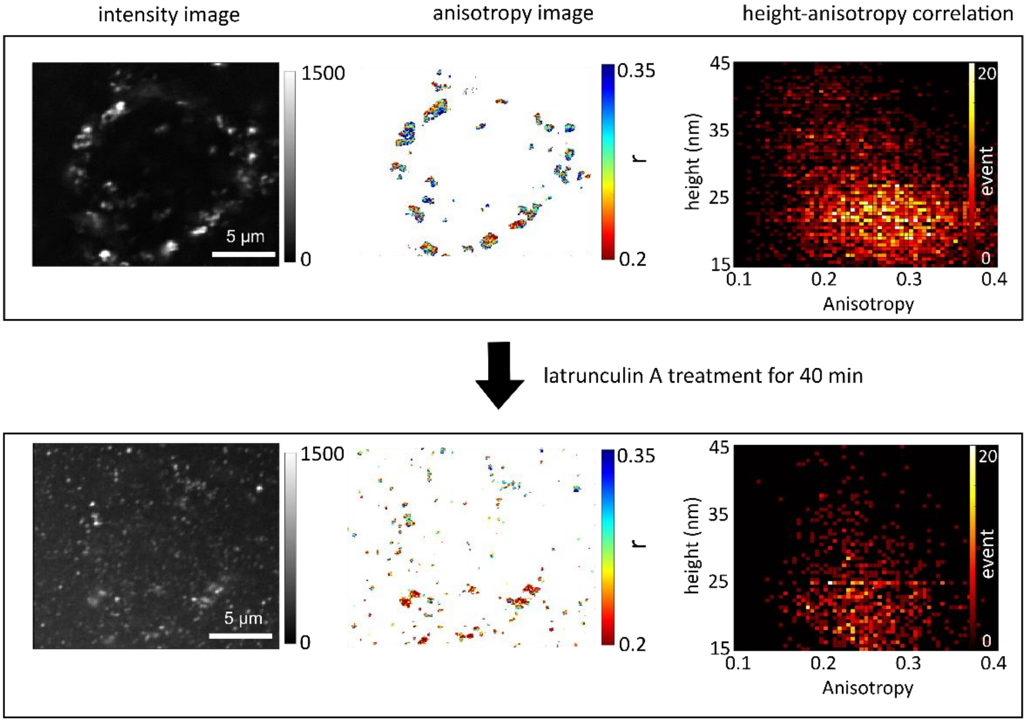

Figure S15. Anisotropy measurements before and after treatment for a cell on MTP-modified MIET substrate. Up: intensity image, anisotropy map, and 2D height-anisotropy correlation histogram before latrunculin A (0.1 µg/mL) treatment. Bottom: the corresponding intensity image, anisotropy map, and 2D height-anisotropy correlation histogram after latrunculin A treatment for 40 min.

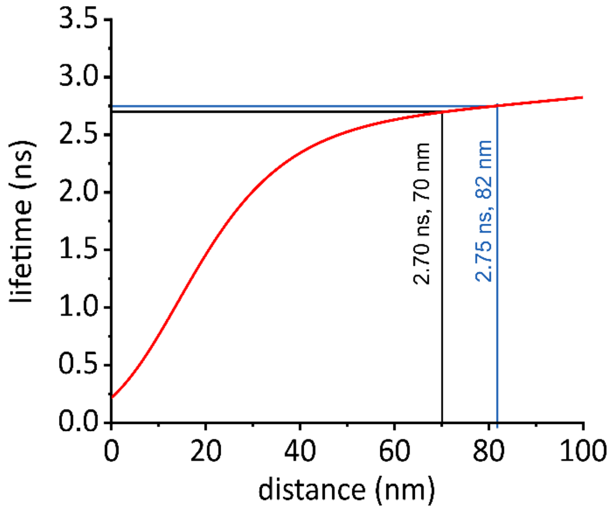

Figure S16. MIET calibration curve for Alexa Fluor® 488–labeled antibodies. Curves were calculated for a dipole emitting at a wavelength of 525 nm with respect to the interface in random orientation. The MIET substrate was fabricated by depositing 10 nm of gold and 15 nm of SiO2 on a cover slide.

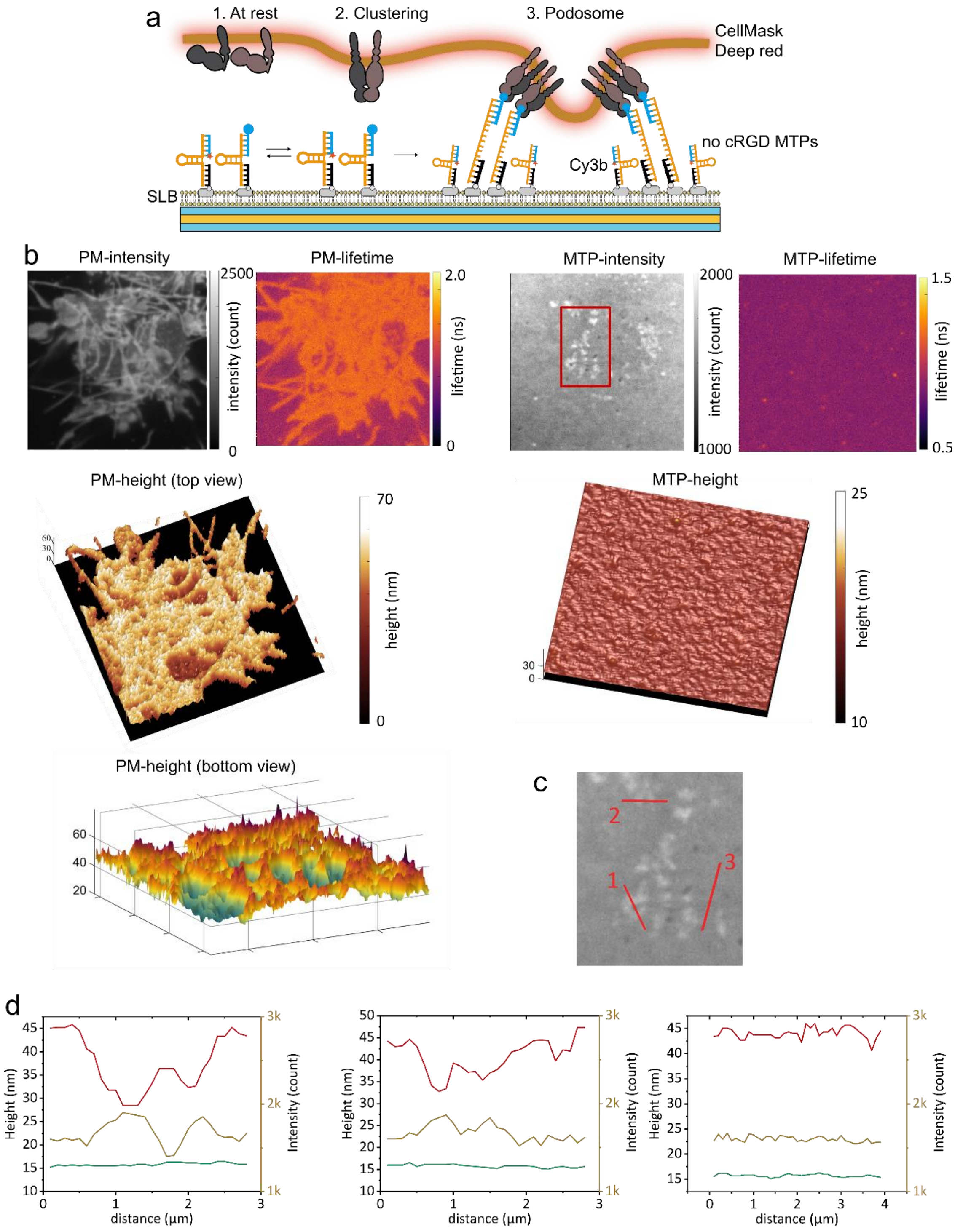

Figure S17. MIET measurement on 4.7 pN Cy3-MTPs without cRGD and RGD-MTPs without Cy3 on SLB. (a) Schematic diagram of the design of the control measurement. (b) Fluorescence intensity images, lifetime images, 3D-reconstructed height maps of the PM and 4.7 pN MTP for a single cell. (c) Enlarged area showing the marks for height profiles and intensity profiles. (d) The height profiles (red curves and green curves) for different lines marked in plane b. The yellow curves are the fluorescence intensity profile of the Cy3-DNA probe. Since the MTP was not modified with the cRGD molecule, which is necessary for integrin binding, no unfolded MTPs were observed in the lifetime or height images.

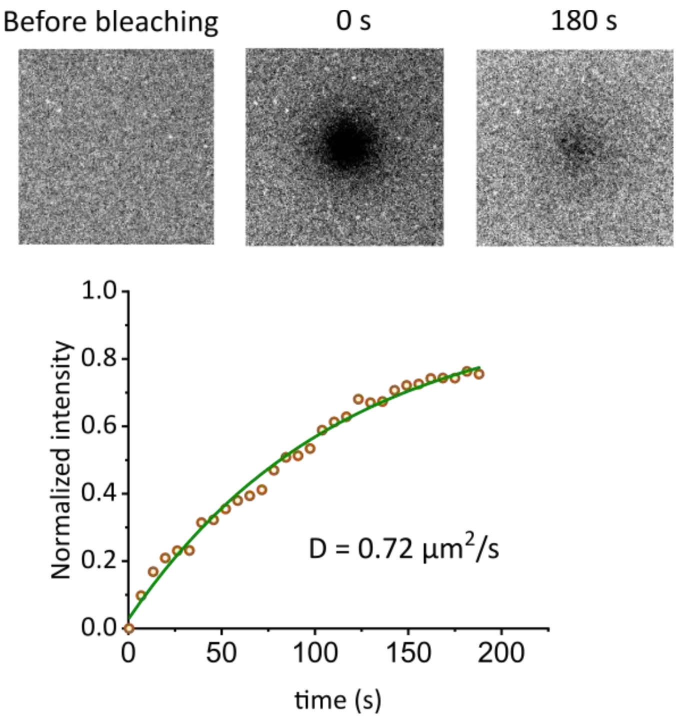

Figure S18. Fluorescence recovery after photobleaching (FRAP) measurement of Cy3- MTP on SLB. TOP: representative images of Cy3-DNA-probes on SLB before photobleaching, immediately after photobleaching, and after recovery. Bottom: fluorescence recovery profile after photobleaching. Data were normalized to the SLB intensity before bleaching. Solid line was fitted with equation $y = A(1 - e^{-bx})$, where A corresponds to the mobile fraction and b is related to the diffusion time $t_{1/2} = ln2/b$. The fitting gives the diffusion coefficient of 0.72 μm^2/s based on the equation, $D = w^2/4t_{1/2}$, where w is the radius of the bleaching area.

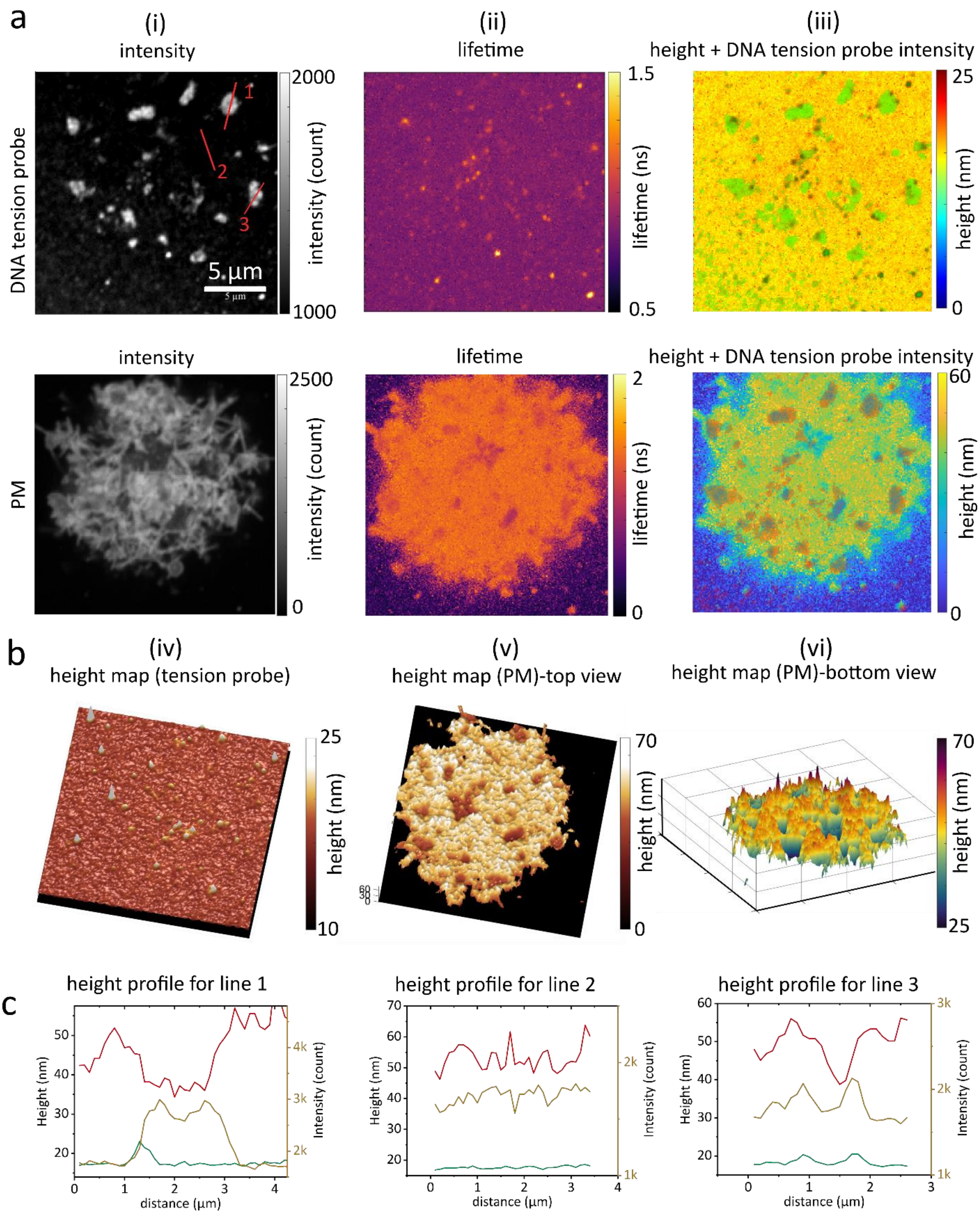

Figure S19. MIET measurement on 19 pN Cy3-MTP on SLB. (a) Fluorescence intensity images (i), lifetime images (ii), and height images (iii) for Cy3-MTP and PM measured from a Cos7 cell. The height images are overlapped with the intensity image of tension probe to show that the podosome regions have higher Cy3-MTP position and lower PM height. (b) The 3D reconstructed height maps for Cy3-MTP and PM. (c) The height profiles (red curves and green curves) for different lines marked in plane a. The yellow curves are the fluorescence intensity profile of the Cy3-MTP.

References

[1] C. Albrecht, *Anal Bioanal Chem* **2008**, *390*, 1223–1224.
[2] D. Magde, R. Wong, P. G. Seybold, *Photochem Photobiol* **2002**, *75*, 327–334.
[3] B. Jo Harvey, C. Perez, M. Levitus, *Photochemical & Photobiological Sciences* **2009**, *8*, 1105–1110.
[4] A. Iqbal, S. Arslan, B. Okumus, T. J. Wilson, G. Giraud, D. G. Norman, T. Ha, D. M. J. Lilley, *Proceedings of the National Academy of Sciences* **2008**, *105*, 11176–11181.
[5] M. E. Sanborn, B. K. Connolly, K. Gurunathan, M. Levitus, *J. Phys. Chem. B* **2007**, *111*, 11064–11074.
[6] J. M. Brockman, A. T. Blanchard, V. Pui-Yan, W. D. Derricotte, Y. Zhang, M. E. Fay, W. A. Lam, F. A. Evangelista, A. L. Mattheyses, K. Salaita, *Nat Methods* **2018**, *15*, 115–118.
[7] A. I. Chizhik, J. Rother, I. Gregor, A. Janshoff, J. Enderlein, *Nature Photon* **2014**, *8*, 124–127.
[8] A. Ghosh, A. I. Chizhik, N. Karedla, J. Enderlein, *Nat Protoc* **2021**, *16*, 3695–3715.
[9] S. J. Shattil, C. Kim, M. H. Ginsberg, *Nat Rev Mol Cell Biol* **2010**, *11*, 288–300.
[10] D. Hanein, N. Volkmann, *Subcell Biochem* **2018**, *87*, 353–363.
[11] M. Maus, M. Cotlet, J. Hofkens, T. Gensch, F. C. De Schryver, J. Schaffer, C. A. M. Seidel, *Anal. Chem.* **2001**, *73*, 2078–2086.
[12] K. Santra, J. Zhan, X. Song, E. A. Smith, N. Vaswani, J. W. Petrich, *J. Phys. Chem. B* **2016**, *120*, 2484–2490.
[13] X. Liu, D. Lin, W. Becker, J. Niu, B. Yu, L. Liu, J. Qu, *J. Innov. Opt. Health Sci.* **2019**, *12*, 1930003.
[14] D. Sakhapov, I. Gregor, N. Karedla, J. Enderlein, *J. Phys. Chem. Lett.* **2022**, *13*, 4823–4830.
[15] R. Glazier, J. M. Brockman, E. Bartle, A. L. Mattheyses, O. Destaing, K. Salaita, *Nat Commun* **2019**, *10*, 4507.
[16] N. Oleksiievets, N. Mougios, D. C. Jans, L. Hauke, J. C. Thiele, S. Basak, S. Jakobs, F. Opazo, J. Enderlein, R. Tsukanov, **2024**, 2024.04.02.587536.
[17] Y. Zhang, C. Ge, C. Zhu, K. Salaita, *Nat Commun* **2014**, *5*, 5167.
[18] A. Blanchard, J. D. Combs, J. M. Brockman, A. V. Kellner, R. Glazier, H. Su, R. L. Bender, A. S. Bazrafshan, W. Chen, M. E. Quach, R. Li, A. L. Mattheyses, K. Salaita, *Nat Commun* **2021**, *12*, 4693.
